# Integrating bulk RNA-seq, Mendelian randomization and single-cell RNA-seq to elucidate the roles of lactate metabolism related markers-SLC25A4 and keratinocyte in the pathogenesis of psoriasis

**DOI:** 10.3389/fimmu.2026.1842933

**Published:** 2026-05-29

**Authors:** Yakun Wang, Xiuwei Wang

**Affiliations:** 1 Department of Dermatology, Venereology and Cosmetology, Beijing Chaoyang Hospital, Capital Medical University, Beijing, China; 2 Translational Medicine Laboratory, Capital Center for Children’s Health, Capital Medical University, Capital Institute of Pediatrics, Beijing, China

**Keywords:** keratinocytes, lactate metabolism, psoriasis, single-cell RNA sequencing, SLC25A4

## Abstract

**Background:**

Aberrant metabolic reprogramming-characterized by enhanced glycolysis and lactate accumulation-contributes to the pathogenesis of psoriasis (PsO). However, lactate metabolism-related genes that causally link mitochondrial dysfunction, immune dysregulation, and keratinocyte (KC) abnormalities in PsO remain insufficiently defined. This study aimed to identify robust lactate metabolism–associated biomarkers and new therapeutic targets in PsO.

**Methods:**

Bulk RNA-seq datasets were integrated with a curated lactate-related gene set to identify differentially expressed lactate-related genes (DE-LRGs) in PsO. Weighted Gene Co-expression Network Analysis (WGCNA), differential expression analysis, and two-sample Mendelian randomization (MR) were applied to prioritize candidate genes with causal associations. Machine learning algorithms were employed to identify robust diagnostic biomarkers and construct an artificial neural network (ANN) model. Functional enrichment, immune infiltration analysis, and immune mediator correlation analyzes were performed. Single-cell RNA sequencing (scRNA-seq) data were analyzed to determine cell-type specificity and KC subtype remodeling. Immunohistochemistry and *in vitro* experiments were conducted to validate the biomarker.

**Results:**

A total of 23 DE-LRGs were identified in PsO. Integrated network analysis and MR prioritized seven DE-LRGs with potential causal relevance. Machine learning approaches identified three robust biomarkers—GOT2, PYGL, and SLC25A4. The ANN model demonstrated excellent diagnostic performance across independent cohorts (AUC > 0.90). Functional enrichment indicated significant involvement in the JAK–STAT signaling pathway and aminoacyl-tRNA biosynthesis. Expression levels of the biomarkers were significantly correlated with immune-cell infiltration, particularly dendritic cells and resting mast cells, as well as key immune mediators. scRNA-seq analysis revealed substantial remodeling of KC subtypes in PsO, with altered differentiation trajectories and disrupted lactate-associated signaling and intercellular communication networks. SLC25A4 exhibited consistent downregulation in PsO at both bulk and single-cell levels, which was confirmed by immunohistochemistry. *In vitro*, SLC25A4 knockdown in HaCaT cells reduced cell viability, while promoting apoptosis. However, this study has certain limitations, as it is primarily based on public databases and *in vitro* experiments, without animal model validation. Further validation through multicenter, prospective cohorts and *in vivo* experiments is required.

**Conclusion:**

This study systematically characterized the landscape of lactate metabolism-related genes in PsO and identified SLC25A4 as robust diagnostic biomarker, highlighting the translational potential of targeting lactate metabolism, particularly SLC25A4, as a diagnostic and therapeutic strategy in PsO.

## 1 Introduction

Psoriasis (PsO) is a chronic inflammatory disease affecting approximately 2% to 3% of the global population (1). It occurs equally in males and females and exhibits a bimodal age of onset, peaking between 20–30 years and 60–70 years (2, 3). PsO manifests in various clinical subtypes, including plaque, guttate, erythrodermic, pustular, inverse, nail, and PsO arthritis (PsA) (4). Topical therapy is the mainstay of treatment for mild-to-moderate PsO. However, over the past two decades, progress in this therapeutic area has remained relatively limited (5). The utility of current topical agents (e.g., corticosteroids, vitamin D analogs, tazarotene, coal tar) is often limited by adverse effects, constraining both their short- and long-term use (6). Importantly, PsO is now recognized not as an isolated skin disorder but as a systemic immune-mediated inflammatory disease driven by Th1/Th17 cell axes and cytokines such as IL-17 and TNF-α. This systemic inflammation is linked to multiple comorbidities, including cardiovascular disease, psoriatic arthritis, and neurological disorders such as Alzheimer’s disease, which shares overlapping inflammatory pathways and oxidative stress mechanisms with PsO (7). Therefore, further elucidation of the pathogenesis of PsO may reveal novel therapeutic targets, which holds significant implications for the development of personalized treatment strategies.

Lactate is now established as a central metabolic hub participating in gluconeogenesis, signal transduction, and immune regulation (8). It serves as a key metabolic orchestrator bridging glycolysis and mitochondrial respiration, functioning as a primary messenger that triggers immediate, short-term, and long-term adaptive cellular responses to ATP supply challenges (9, 10). In immune cells, lactate modulates metabolism, immune responses, and gene/protein function via lactylation (8, 11, 12). In PsO, lactate levels correlate with disease severity (13), reprogram immune cells to sustain inflammation (14), and four lactate metabolism-associated genes (STAT1, HK2, CXCL1, ALOX12B) serve as diagnostic biomarkers (15), establishing lactate metabolism as a pivotal therapeutic target. Recent single-cell transcriptomic studies have further revealed that PsO lesions exhibit remodeling of keratinocyte subtypes and altered intercellular communication networks, with key metabolic pathways such as oxidative phosphorylation and reactive oxygen species signaling being significantly dysregulated (16). These findings underscore the fundamental role of lactate metabolism in PsO pathogenesis, positioning it as a promising target for novel preventive and therapeutic strategies.

Mendelian randomization (MR) employs genetic variants as instrumental variables to support causal inference between exposures and outcomes while minimizing confounding (17, 18). In PsO research, MR has identified causal links between circulating metabolite levels and disease risk (19) and between PsO and cardiovascular disease or specific immune cell populations (20, 21). Separately, single-cell RNA sequencing (scRNA-seq) provides whole-transcriptome profiling at single-cell resolutions (22, 23). In PsO, it has revealed the heterogeneity of keratinocytes and fibroblasts (24), and identified disease-associated CD8^+^ T-cell subsets along with the immunoregulatory marker G3BP2 (25, 26). In addition, a marked increase in the frequency of CD4^+^ T cells was observed within PsO lesions (16). Keratinocytes function not merely as passive targets but as active drivers of innate immune responses and inflammation, partly through the UBE2L3/IL-1β/STAT3 axis and the CXCL16/CXCR6 signaling network (27). Together, these approaches could advance causal understanding of PsO pathogenesis at the cellular level.

In this study, we performed an integrated analysis of transcriptome sequencing to identify lactate metabolism–related biomarkers in PsO. Candidate genes were screened by differential expression analysis and weighted gene co-expression network analysis (WGCNA), followed by MR to verify the causal relationships between genes and PsO. Subsequently, three machine-learning algorithms were applied to identify lactate metabolism–related biomarkers. Gene set enrichment analysis (GSEA), immune infiltration analysis, regulatory network construction, scRNA-seq analysis, molecular docking, and *in vitro* cell experiments were performed to investigate the molecular mechanisms and potential therapeutic value of these biomarkers, thus providing a foundation for developing targeted therapeutic interventions.

## 2 Methods

### 2.1 Data acquisition

In the study, 5 PsO-related datasets (GSE54456, GSE13355, GSE14905, GSE182740, GSE220116) were retrieved from the Gene Expression Omnibus (GEO) database (http://www.ncbi.nlm.nih.gov/geo/). Specifically, GSE54456 (platform: GPL9052) contained 90 PsO lesional and 81 control skin samples (28); GSE13355 and GSE14905 (both platform: GPL570) included 58 PsO/64 controls (29); and 33 lesional/21 controls (30); respectively; GSE182740 (platform: GPL570) had 9 lesional and 6 controls (31); and GSE220116 (scRNA-seq, platform: GPL18573) comprised 11 pre-treatment lesional and 10 controls (32). Additionally, nine lactate-associated gene sets (e.g., “GOBP_LACTATE_METABOLIC_PROCESS”) were searched via the keyword “Lactate” from the Molecular Signatures Database (MSigDB) (https://www.gsea-msigdb.org/gsea/msigdb/), covering metabolism, transport, enzyme activity and related phenotypes. Genes from these sets were extracted, integrated and deduplicated to obtain 350 lactate metabolism-related genes (LRGs) (**Supplementary Table 1**).

Genome-wide association study (GWAS) summary statistics for exposure factors were collected from the Integrative Epidemiology Unit-Open Genome Wide Association Studies (IEU OpenGWAS) database, with only cis-expression quantitative trait loci (cis-eQTLs) downloaded. The PsO (outcome variable) GWAS dataset was downloaded from IEU OpenGWAS using “PsO” as the keyword; dataset “ebi-a-GCST90038681” included 5,427 European cases, 479,171 European controls, and 9,587,836 single nucleotide polymorphisms (SNPs).

To identify genes closely associated with PsO, Weighted Gene Co-expression Network Analysis (WGCNA) was performed using the WGCNA (V 1.72-5) package (33) on all GSE54456 samples, with sample expression profiles as traits. First, to assess inter-sample correlation in GSE54456, the absolute deviation (mad) of each gene was calculated via the hclust function, followed by hierarchical clustering of samples based on the expression matrix to determine outlier removal needs.

To ensure gene interactions fit the scale-free distribution, weighted correlation coefficients (gene correlation coefficients raised to the Nth power) were used for soft threshold determination. Suitable soft threshold power (1–20) was selected by analyzing relationships between soft threshold β and scale-free network R² (set at 0.80), as well as average connectivity, screening for values exceeding the red cutoff with connectivity near 0.

Using the dynamic tree cutting algorithm (minimum 100 genes per module, merging threshold 0.3), a scale-free network was constructed with the screened soft threshold, and genes were clustered into color-labeled modules. Pearson correlation analysis was conducted to link modules with sample expression phenotypes, calculating the module eigengene-phenotype correlation matrix and plotting a heatmap (|cor| > 0.30, *P* < 0.05) via the labeled heatmap function. The module most strongly correlated with sample expression profiles was designated the key module, and its genes were extracted as PsO-related module genes (PRMGs).

### 2.2 Identification, functional enrichment and protein-protein interactions of differentially expressed LRGs

PsO-related differentially expressed genes (PsO-DEGs) in GSE54456 were obtained via the DESeq2 (V 1.42.0) package (34) (*P*.adjust < 0.05, |log2 fold-change (FC)| > 0.5). A volcano plot was generated with ggplot2 (V 3.5.1) (https://link.springer.com/book/10.1007/978-0-387-98141-3), and a heatmap (top 10 up/down-modulated genes by log2FC) with ComplexHeatmap (V 2.18.0) (35). PsO-DEGs, PRMGs, and LRGs were intersected using ggvenn (V 0.1.10) (https://CRAN.R-project.org/package=ggvenn) to get DE-LRGs, which were converted from SYMBOL to ENTREZID via org.Hs.eg.db (V 3.18.0) (36). Gene Ontology (GO) and Kyoto Encyclopedia of Genes and Genomes (KEGG) enrichment analyzes were performed with clusterProfiler (V 4.10.1) (37). Protein-protein interaction (PPI) networks (confidence > 0.4) were constructed via the search tool for the retrieval of interacting genes (STRING) database (http://www.string-db.org/) and the Cytoscape (V 3.9.1) software (38), then screened by the Maximum Neighborhood Component (MNC) algorithm.

### 2.3 Mendelian randomization analysis

To clarify the causal relationship between DE-LRGs and PsO, MR analysis was performed with DE-LRGs as exposures, PsO as the outcome, and SNPs as instrumental variables (IVs) using the TwoSampleMR (V 0.6.4) package (39). MR relied on three assumptions: IVs were closely linked to exposures, unrelated to confounders, and influenced outcomes only via exposures. IVs were screened by: ① *P* < 5×10^-8^, ≥ 3 SNPs per exposure; ② eliminating LD SNPs (R² < 0.001, kb = 10, clump = TRUE); ③ removing SNPs associated with PsO from PhenoSPsoriasisnner (http://www.phenosPsoriasisnner.medschl.Psoriasism.ac.uk); ④ excluding IVs with F-statistic < 10 (40). The 5 algorithms (MR Egger (41), weighted median (42), inverse variance weighted (IVW) (43), simple mode (44), and weighted mode (45)) were used via harmonise_data; candidate exposures were selected by IVW (*P* < 0.05), with odds ratio (OR) > 1 as risk factors and OR < 1 as protective factors.

To clarify exposure-outcome associations and MR analysis reliability, three analyzes were performed. First, correlation scatter plots (ggplot2, V 3.5.1) integrated SNP-exposure/outcome effects, with slopes indicating risk/protective roles and intercepts confounding. Second, mr_forest_plot assessed each SNP’s effect, with the IVW model’s overall effect (red line) showing diagnostic utility. Finally, mr_funnel_plot verified compliance with randomization/Mendel’s second law via symmetrical SNP distribution.

To verify the reliability of MR analysis results, sensitivity analyzes (heterogeneity, horizontal pleiotropy, leave-one-out tests) and an MRSteiger directionality test were performed. (1) Heterogeneity was assessed via the mr_heterogeneity function: Q-pval > 0.05 indicated no heterogeneity (fixed-effects IVW model), while Q-pval < 0.05 indicated heterogeneity (random-effects IVW model). (2) Horizontal pleiotropy was tested using mr_pleiotropy_test, MR-Egger, and MR-PRESSO; *P* > 0.05 confirmed no pleiotropy and reliable results. (3) The leave-one-out test (mr_leaveoneout function) excluded IVs sequentially to evaluate outcome stability—minimal effect changes indicated robust results (IVW model overall effect: red line). Additionally, the directionality_test function implemented the MRSteiger test; TRUE results with *P* < 0.05 validated correct causal direction. Hub genes were defined as candidate exposures causally linked to PsO that passed all tests.

### 2.4 Machine learning analysis

In order to obtain the characteristic genes by machine learning, the least absolute shrinkage and selection operator (LASSO) logistic regression analysis was executed based on the hub genes in GSE54456 dataset with the help of the glmnet (V 4.1-8) package (46), and 10-fold cross validation was performed. The hub genes in the GSE54456 dataset were subjected to support vector machine-recursive feature elimination (SVM-RFE) by means of the e1071 (V 1.7-14) package (https://CRAN.R-project.org/package=e1071). The optimal variables were investigated by removing the character vectors generated by SVM. Meanwhile, the importance of hub genes in the GSE54456 dataset was ranked using the Boruta algorithm based on the Boruta (V 8.0.0) package (47), and the confirmed genes obtained were retained. The intersection using ggvenn (V 0.1.10) package of genes in LASSO, SVM-RFE, and Boruta was utilized to obtain the characteristic genes.

### 2.5 Identification of biomarkers

To clarify the expression levels of characteristic genes in datasets GSE54456, GSE13355, GSE14905, and GSE182740, Wilcoxon tests were performed to compare their expression between PsO and control samples across these four datasets. Characteristic genes with significant expression differences and consistent trends across all four datasets were selected as candidate biomarkers. Subsequently, receiver operating characteristic (ROC) curve analysis (pROC, V 1.18.5) (48) evaluated their discriminatory capacity in GSE54456 and GSE13355; candidates with AUC > 0.7 in both datasets were designated as formal biomarkers. Additionally, artificial neural network (ANN) models were constructed using the neuralnet (V 1.44.2) package (https://CRAN.R-project.org/package=neuralnet) for predictive value assessment, with GSE54456 as the training set and the other three datasets as validation sets; AUC > 0.7 confirmed good diagnostic efficacy for PsO.

### 2.6 Gene set enrichment analysis and gene-gene interaction network

To clarify the biological functions of biomarkers, Spearman correlation analysis was performed on GSE54456’s PsO and control samples using the psych (V 2.4.3) package (https://CRAN.R-project.org/package=psych). After sorting correlation coefficients in descending order, GSEA was conducted via clusterProfiler (V 4.10.1), with the MSigDB (https://www.gsea-msigdb.org/gsea/msigdb) “c2” dataset (C2: CP: KEGG) from msigdbr (V 7.5.1) (https://CRAN.R-project.org/package=msigdbr) as the reference (|normalized enrichment score (NES)| > 1, false discovery rate (FDR) < 0.25, and *P* < 0.05). Additionally, a gene-gene interaction (GGI) network was constructed in GeneMANIA (https://genemania.org/, Homo sapiens) to explore biomarkers’ functional status and associated genes.

### 2.7 Localization, correlation and disease prediction analyzes

To explore tissue-specific differences of biomarkers, their tissue expression levels were retrieved from the Human Protein Atlas (HPA) (https://www.proteinatlas.org/), and tissue-level expression scores from Bgee (https://bgee.org). Spearman correlation analysis (cor function) was performed on GSE54456’s PsO/control samples (|cor| > 0.3, *P* < 0.05). Additionally, biomarkers were input into the Comparative Toxicogenomics Database (CTD) (http://ctdbase.org/), with top 5 related diseases (sorted by Inference Score) selected for visualization.

### 2.8 Immune cell infiltration and immune factors analyzes

To characterize the PsO immune microenvironment, infiltration levels of 22 immune cells (49) in GSE54456 (PsO vs. control) were computed via CIBERSORT (V 0.1.0) (50). Samples with *P* > 0.05 were filtered out, and differential immune cells (DICs) were identified by Wilcoxon test (*P* < 0.05). Spearman correlation analysis (psych, V 2.4.3) was performed for DIC-DIC and DIC-biomarker relationships (|cor| > 0.3, *P* < 0.05). Top correlated IFs, DICs, and biomarkers were used to construct an association network via Cytoscape (V 3.9.1).

### 2.9 Regulatory networks analysis

The miRNA-biomarker interactions were explored via the multiMiR (V 0.98.0.2) package (51),. Biomarker-targeting miRNAs were predicted using DIANA-microT (http://diana.imis.athena-innovation.gr/DianaTools/index.php) and ELMMo (https://mirz.unibas.ch/ElMMo3/index.php) in the get_multimir function, with overlapping key miRNAs extracted via ggvenn (V 0.1.10). miRNet (https://www.mirnet.ca/) predicted lncRNAs (≥10 interactions) and circRNAs (≥30 interactions) binding key miRNAs. Cytoscape (V 3.9.1) constructed lncRNA/circRNA-miRNA-mRNA networks. JASPAR (https://jaspar.elixir.no/) within NetworkAnalyst (https://www.networkanalyst.ca/) predicted TFs, and a TF-mRNA network was built with Cytoscape.

### 2.10 Drug prediction and molecular docking analyzes

To identify biomarker-targeting therapeutic agents, potential drugs/molecular compounds were retrieved from the Drug-Gene Interaction database (DGIdb, https://dgidb.org/) and Drug Signatures Database (DSigDB, https://dsigdb.tanlab.org/DSigDBv1.0/). The top-scoring agent per biomarker (sorted by interaction score) was selected for molecular docking. For binding validation, chemical structures (SDF files) of key agents were obtained from PubChem (https://pubchem.ncbi.nlm.nih.gov/), and 3D protein structures (PDB files) of biomarkers from RCSB PDB (https://www.rcsb.org/). CB-Dock2 (https://cadd.labshare.cn/cb-dock2/php/index.php) performed docking simulations; binding energy ≤ -5 kcal/mol indicated stable binding.

### 2.11 scRNA-seq analysis

The GSE220116 dataset was processed using the Seurat (V 5.1.0) package (52). First, batch integration was conducted via the harmony function, followed by sample data quality control. High-quality cells and genes were selected with the criteria: 200 ≤ nFeature RNA ≤ 3000, nCount RNA < 20000, and percent.mt < 15%. After normalizing the filtered single-cell data using the NormalizeData function, highly variable genes (HVGs) were identified by the variance-stable transformation (vst) method of the FindVariableFeatures function, and the top 10 HVGs were marked via the LabelPoints function. Subsequently, HVGs were scaled by the ScaleData function, and principal component analysis (PCA) was performed on the scaled data using the RunPCA function. PC significance (*P* < 0.05) was quantified via the ScoreJackStraw function after calculating PC P-values with the Jackstraw-based permutation test, and results were visualized by the Elbowplot function. Unsupervised clustering was implemented using the FindClusters and FindNeighbors functions (resolution = 0.7, dimension = 30), with results visualized via uniform manifold approximation and projection (UMAP) using the RunUMAP function. Marker genes from the literature (53) and (54) were used for cell type annotation. Bubble plots and bar charts exhibited marker gene expression levels and cell type proportions in PsO and control samples, respectively. Functional enrichment of cell types was analyzed using the ReactomeGSA (V 1.16.1) package (55), and key cell subtypes were subclustered, annotated, and analyzed based on literatures (53) and (54). To further characterize the immune microenvironment, T cells and fibroblasts were extracted separately and subjected to independent subclustering analyzes. For T cell subclustering, extracted T cells were re-embedded using PCA dimensionality reduction, batch effects were corrected via the Harmony algorithm, and unsupervised clustering was performed using FindNeighbors and FindClusters functions (dims = 20, resolution = 0.1). For fibroblast subclustering, the same pipeline was applied (dims = 15, resolution = 0.1). Subclusters were annotated based on canonical marker genes from published literature (56, 57). t-SNE plots were used to visualize subtype distribution, bubble plots illustrated marker gene expression across subclusters, and bar charts displayed subtype proportional differences between PsO and control groups.

### 2.12 Identification of differentially expressed cells and key cells

To compare cell type abundance between PsO and control groups in the scRNA-seq dataset, the Wilcoxon test was performed. Screened differential cell types (DE-cells) with significant biomarker expression differences were defined as key cells. Bubble and UMAP plots were used to show biomarker expression in cell types and key subtypes of both groups.

### 2.13 Cell communication analysis

To analyze interactions between key cells and other annotated cell types, molecular interactions in PsO and control specimens from the GSE220116 dataset were separately analyzed using the CellChat (V 1.6.1) package (58). Bubble plots illustrated ligand-receptor interactions in key cells and their subtypes (*P* < 0.05). Key cells and subclusters were scored for S, G2, and M phases via CellCycleScoring, using Seurat’s cell cycle marker genes. Functional enrichment of key cell subtypes was analyzed by the ReactomeGSA (V 1.16.1) package (55)’s analyze_sc_clusters function. Average gene expression per subtype was mapped to Reactome pathways, and significantly different pathways were screened and visualized via the package’s plot_gsva_heatmap function.

### 2.14 Pseudotime trajectory analysis

To explore key cell trajectories, key cells from the GSE220116 dataset were subjected to pseudotime trajectory analysis using the monocle (V 2.30.1) package (59). Key cells were dimensionally reduced via the DDRTree algorithm, projected onto a root and two branches, and clustered into subgroups by the clusterCells function. Density curves were plotted with the ggpubr (V 0.6.0) package (https://CRAN.R-project.org/package=ggpubr) to show cell number changes across developmental phases. Biomarker expression trends were analyzed using the plot_pseudotime_heatmap function.

### 2.15 Immunohistochemistry analysis

Ten patients with plaque PsO and 10 controls of healthy skin samples were recruited from the Department of Dermatology, Venereology and Cosmetology of Beijing Chaoyang Hospital. Patients with a diagnosis of plaque PsO, confirmed by both clinical manifestations and histopathological biopsy, and with active PsO lesions of more than one year’s duration, were enrolled in the study. Subjects with other subtypes of PsO, allergic diseases, or other dermatological disorders were excluded. To minimize the potential influence of prior therapy on tissue protein expression, PsO patients were required to have received no topical or systemic antipsoriatic treatment for at least 2 months before enrollment. Tissue specimens from patients were initially heated at 65 °C for 2 hours to ensure proper adherence. Subsequent dewaxing and rehydration procedures were performed using xylene and gradient ethanol solutions, respectively. Antigen retrieval was achieved by immersing the samples in EDTA buffer (pH 8.0) and boiling for 20 minutes to expose epitope sites. To minimize non-specific binding, tissue sections were pre-incubated with 2% goat serum. Primary antibody incubation was conducted overnight at 4 °C with the following antibodies: PYGL Polyclonal antibody (Proteintech, catalog no. 15851-1-AP) at a dilution of 1:400; GOT2 antibody [EPR12354(B)] (Abcam, catalog no. ab171739) at a dilution of 1:8000; and SLC25A4 Antibody (Affinity, catalog no. DF6674) at a dilution of 1:200.

### 2.16 Cell culture

The human keratinocyte cell line HaCaT was cultured in high-glucose DMEM supplemented with 10% fetal bovine serum (FBS) and 1% penicillin-streptomycin (Thermo Fisher Scientific). For each transfection, a specific amount of SLC25A4-targeting siRNA (or a non-targeting scrambled siRNA as a negative control) was diluted in a serum-free medium. siRNA was complexed with Lipofectamine RNAiMAX transfection reagent according to the manufacturer protocol. Knockdown efficiency was assessed by western blotting at 72 hours after transfection.

### 2.17 Cell viability assay

Cell viability was assessed using the Cell Counting Kit-8 (CCK-8; Dojindo Molecular Technologies, Inc., Japan) according to the manufacturer’s instructions. The absorbance of each well was measured at 450 nm, utilizing a microplate reader. Data are presented as the mean ± standard deviation (SD).

### 2.18 EdU assay

Cells were seeded at 5 × 10^4^ cells per well in a confocal dish and, after transfection, incubated with 10 µM EdU for 2 hours under 37 °C, 5% CO_2_. Following fixation with 4% paraformaldehyde and permeabilization with 0.5% Triton X-100, the cells were subjected to the Click-iT reaction, counterstained with Hoechst 33342, and imaged using a confocal microscope (Leica TCS SP5 X, Germany).

### 2.19 Flow cytometry

Following transfection, cells were collected, washed with cold PBS, and resuspended in 100 µL of 1× Annexin V binding buffer. They were then stained with a mixture of FITC-Annexin V and Propidium Iodide (5 µL each) for 15 minutes in the dark at room temperature, diluted with an additional 400 µL of 1×Annexin V binding buffer, and analyzed by flow cytometry (BD).

### 2.20 Statistical analysis

Statistical computations were carried out using the R (V 4.3.1) software. The scRNA-seq analysis was carried out with the Seurat (V 5.1.0) package. Student’s *t*-tests were used to compare the two groups. Statistical significance was assessed at a significance level of *P* < 0.05.

## 3 Results

### 3.1 Identification of 23 DE-LRGs in PsO

**Figure 1:**
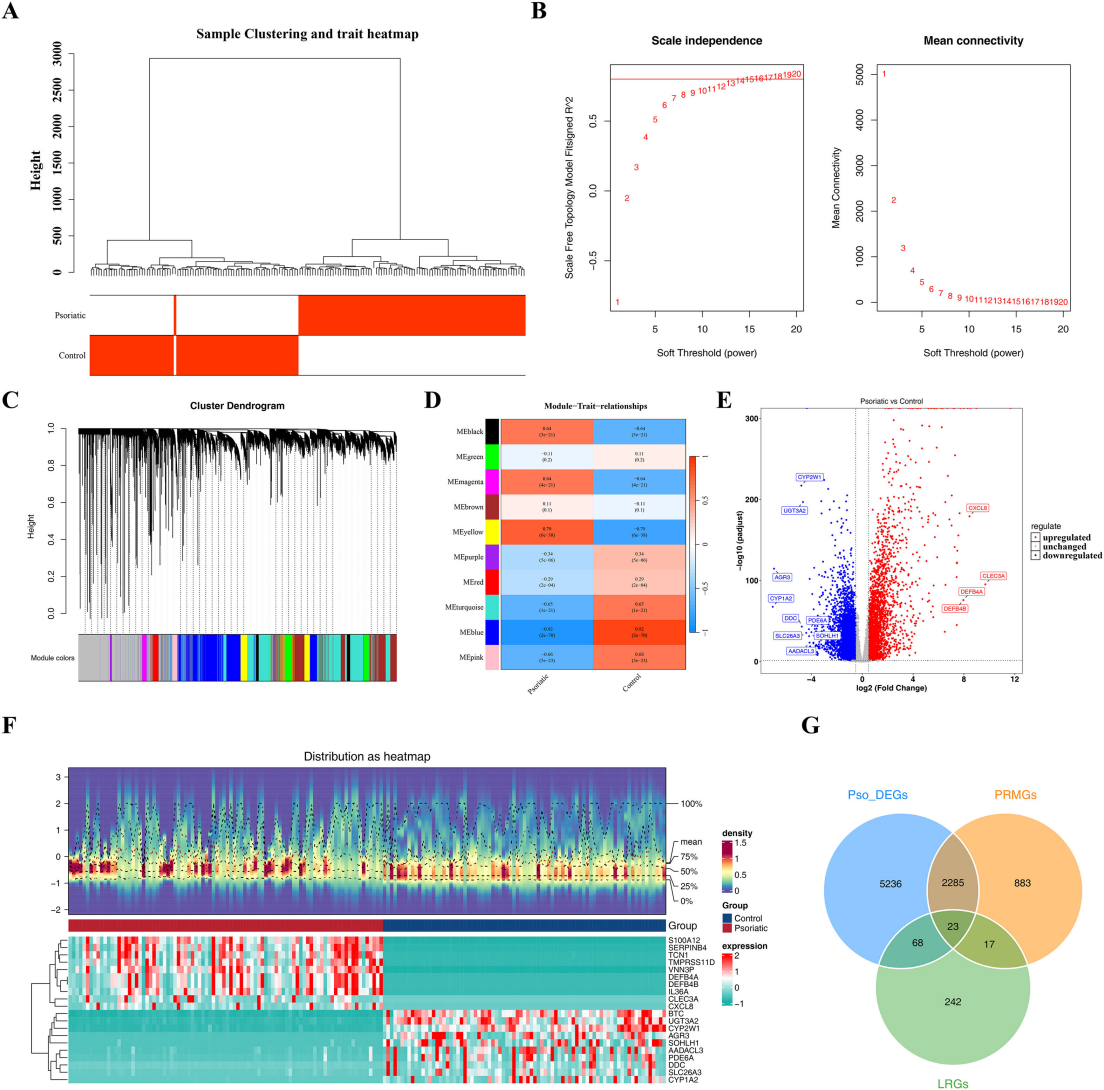
WGCNA analysis. **(A)** Sample hierarchical clustering tree diagram. **(B)** Soft threshold screening diagram. **(C)** Co-expression module screening diagram. The upper half is the hierarchical clustering tree diagram of genes, and the lower half is the gene module. **(D)** Correlation heat diagram of modules and phenotypes. **(E)** Volcanic diagram of DEGs, red represents up-regulation, blue represents down-regulation. **(F)** Heat diagram of DEGs, red expression is higher, blue expression is lower. **(G)** Venn diagram of gene intersection.

First, the clustering of WGCNA indicated the absence of any outlier samples in the GSE54456 dataset (**Figure 1A**). The value of β was 16 based on R^2^ = 0.80 and mean connectivity of 0 (**Figure 1B**). The analysis identified 10 co-expression modules, among which the MEbule module exhibited the highest absolute value of correlation with the phenotype (cor = -0.92, *P* = 2e-70) (**Figures 1C, D**). The MEbule module were recorded as the key module, which contained 3,208 module genes as PRMGs. Second, a total of 7,612 PsO-DEGs (3,073 up-regulated and 4,539 down-regulated) were identified between PsO and control in the GSE54456 dataset (**Supplementary Table 2**). A volcano plot was used to label the top 10 up/down-regulated PsO-DEGs ranked by log2FC(**Figure 1E**). Their expression was shown in the heatmap (**Figure 1F**). Subsequently, 23 DE-LRGs were identified by intersecting the 7,612 PsO-DEGs, 3,208 PRMGs and 350 LRGs (**Figure 1G**).

### 3.2 GO, KEGG, and PPI of 23 DE-LRGs

To further explore the biological function of 23 DE-LRGs, enrichment analysis was performed. The 23 DE-LRGs were enriched in 53 GO terms (*P*.adjust < 0.05), including 33 biological processes (BPs), 1 cellular component (CC), 19 molecular functions (MFs), and 17 KEGG pathways (*P* < 0.05) (**Supplementary Figures 1A, B**; **Supplementary Tables 3**, **4**). Key terms included deoxyribonucleoside metabolism in BP, mitochondrial matrix in CC, vitamin binding in MF, and nucleotide metabolism in KEGG pathways. Additionally, the PPI network contained 15 genes and 15 interaction pairs, with PYGL-LDHA, GOT2-ACACB, and SLC25A4-TK2 interactions identified (**Supplementary Figure 1C**).

### 3.3 Identification of 7 hub genes in PsO

**Figure 2:**
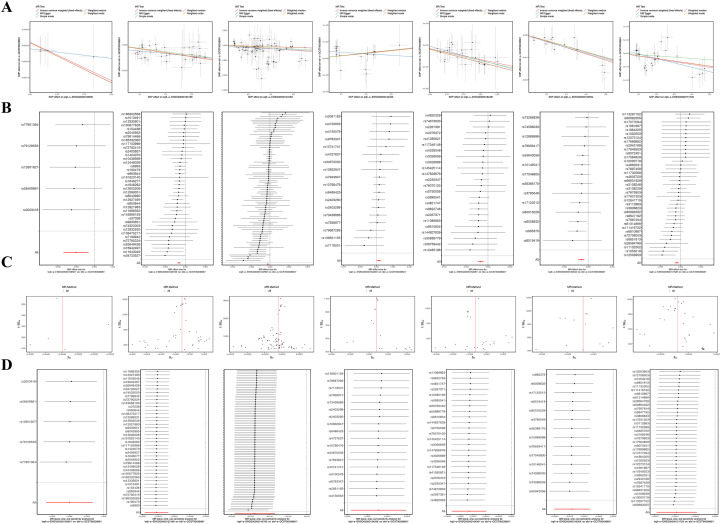
Mendelian randomization analysis. **(A)** Correlation analysis plot between exposure factors and outcomes. **(B)** MR random forest plot. **(C)** MR analysis funnel plot. **(D)** Forest plot of the stepwise exclusion test for candidate exposure factors.

To screen DE-LRGs causally related to PsO, 7 candidate exposure factors (GOT2, PYGL, SLC25A4, C1QBP, LDHA, PLA2G6, KCNN4) were identified via IVW-dominated MR analysis (*P* < 0.05, **Table 1**). LDHA was a PsO risk factor (OR = 1.00079), while the others were protective factors (OR < 1). Scatter and forest plots confirmed consistency with IVW results (**Figures 2A, B**). Funnel plots showed symmetric SNP distribution (**Figure 2C**). Heterogeneity (*P* > 0.05) (**Supplementary Table 5**) and horizontal pleiotropy (*P* > 0.05) (**Supplementary Table 6**) were absent. Leave-one-out analysis verified robust results (**Figure 2D**). MR Steiger test confirmed valid causal associations (TRUE, *P* < 0.05) (**Table 2**). Thus, these 7 factors were defined as hub genes for subsequent analyzes.

**Table 1:** MR analysis of the IVW algorithm.

Symbol	Id. exposure	Method	Nsnp	Pval	Or
GOT2	eqtl-a-ENSG00000125166	Inverse variance weighted	39	2.01E-16	0.998456547
PYGL	eqtl-a-ENSG00000100504	Inverse variance weighted	14	6.36E-06	0.998595691
SLC25A4	eqtl-a-ENSG00000151729	Inverse variance weighted	37	0.000189377	0.999047522
C1QBP	eqtl-a-ENSG00000108561	Inverse variance weighted	5	0.003690087	0.996022890
LDHA	eqtl-a-ENSG00000134333	Inverse variance weighted	18	0.025839679	1.000786304
PLA2G6	eqtl-a-ENSG00000184381	Inverse variance weighted	24	1.68E-06	0.998352541
KCNN4	eqtl-a-ENSG00000104783	Inverse variance weighted	83	4.62E-07	0.999448485

**Table 2:** MR Steiger test.

Id. exposure	Id. outcome	Snp_r2.exposure	Snp_r2.outcome	Correct_causal_direction	Steiger_pval
eqtl-a-ENSG00000125166	ebi-a-GCST90038681	0.32959683761375	0.000190889782748537	TRUE	0
eqtl-a-ENSG00000100504	ebi-a-GCST90038681	0.113632729906957	5.1459255724763e-05	TRUE	0
eqtl-a-ENSG00000151729	ebi-a-GCST90038681	0.156338927551825	9.11434150655273e-05	TRUE	0
eqtl-a-ENSG00000108561	ebi-a-GCST90038681	0.00645125026334706	1.85338766828107e-05	TRUE	3.01383990146598e-35
eqtl-a-ENSG00000134333	ebi-a-GCST90038681	0.106504111763296	3.38254628369246e-05	TRUE	0
eqtl-a-ENSG00000184381	ebi-a-GCST90038681	0.101827830114423	6.90859806579108e-05	TRUE	0
eqtl-a-ENSG00000104783	ebi-a-GCST90038681	0.840278505754183	0.000287331468543069	TRUE	0

### 3.4 Identification of 3 biomarkers in PsO

**Figure 3:**
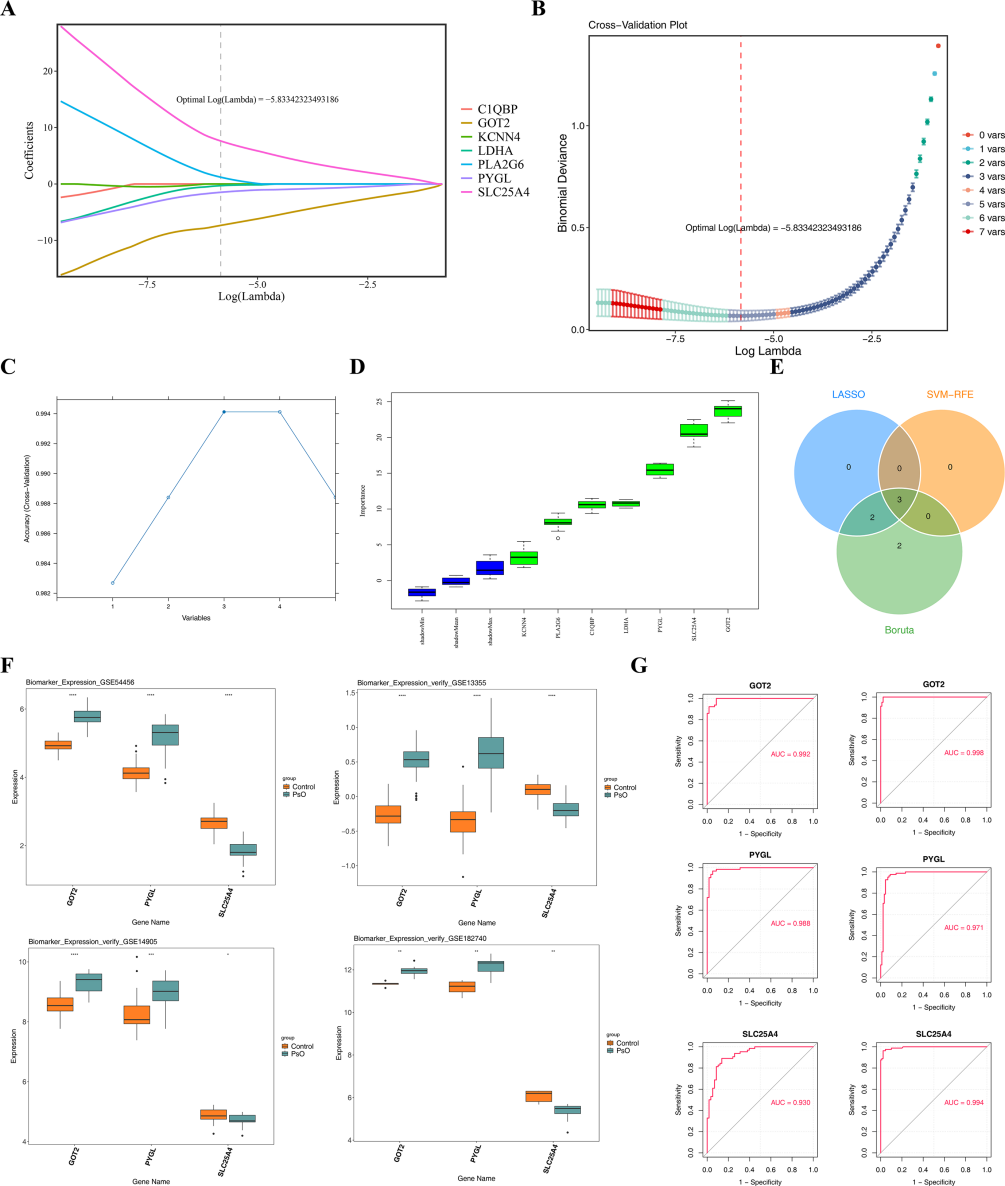
Validation of feature genes and biomarkers screened by machine learning. **(A)** Coefficient screening plot of LASSO analysis. **(B)** Cross-validation plot of LASSO analysis. **(C)** Analysis results of SVM-RFE model. **(D)** Analysis results of Boruta algorithm. **(E)** Venn diagram of overlapping genes in machine learning. **(F)** Box plot for validation of expression levels in different datasets, *P < 0.05, **P < 0.01, ***P < 0.001, ****P < 0.0001. **(G)** ROC curves of biomarkers GOT2, PYGL and SLC25A4 in the training set and validation set.

To further screen PsO-related causal hub genes, LASSO regression with 10-fold cross-validation (lambda.min = 0.002928036) was performed on the GSE54456 dataset, identifying 5 hub genes (GOT2, PYGL, SLC25A4, LDHA, PLA2G6) (**Figures 3A, B**). The SVM-RFE model achieved peak prediction accuracy with 3 genes (GOT2, PYGL, SLC25A4) (**Figure 3C**), while the Boruta algorithm confirmed 7 genes (**Figure 3D**). The 3 overlapping genes (GOT2, PYGL, SLC25A4) were designated characteristic genes (**Figure 3E**). Wilcoxon tests revealed their differential expression between PsO and control samples across four datasets (*P* < 0.05): GOT2 and PYGL were up-regulated, and SLC25A4 down-regulated in PsO (**Figure 3F**). ROC analysis verified their high diagnostic value (AUC > 0.9) in two datasets (**Figure 3G**), thus they were selected as biomarkers for subsequent analyzes.

### 3.5 ANN models and biological pathways of 3 biomarkers in PsO

**Figure 4:**
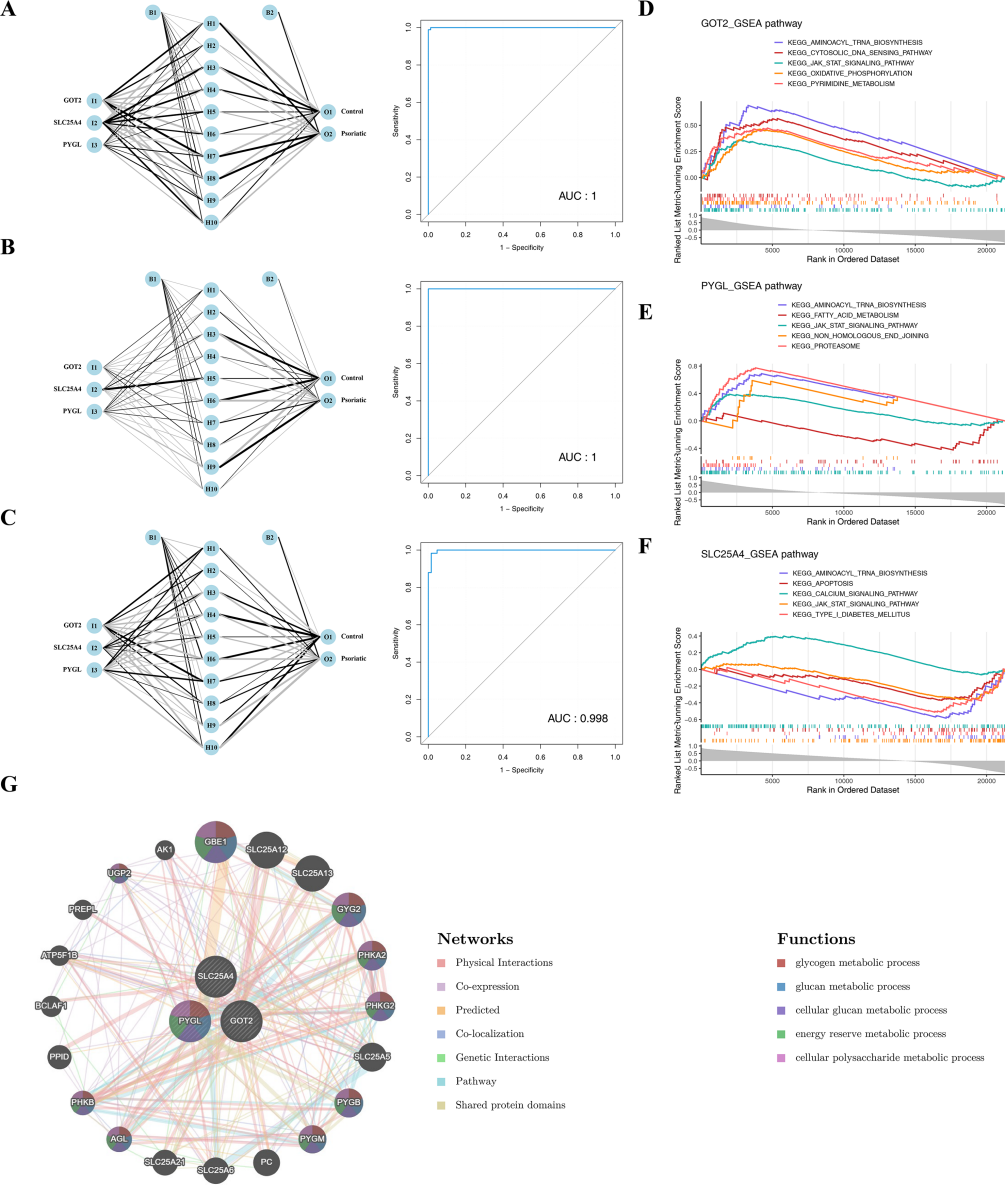
Construction of artificial neural network diagnostic model and GSEA enrichment analysis. Construction and ROC validation of the ANN model in the training set **(A)** and validation sets **(B–D)**. GSEA enrichment results of genes GOT2 **(E)**, PYGL **(F)** and SLC25A4 **(G)**. The figure is divided into 3 parts. The top part is the enrichment score line chart. Each line represents a pathway, and the peak value of each line is the enrichment score of that pathway. The genes before the peak value are the core genes under the gene set of that pathway. The second part marks the genes located in the gene set with lines. The third part is the rank value distribution of all genes. **(H)** GeneMANIA analysis.

ANN models were built using 3 biomarkers to forecast the prevalence of PsO in patients. Notably, in the GSE54456, GSE13355, and GSE14905 datasets, the ANN models constructed by GOT2, PYGL, and SLC25A4 showed good performance, and the AUC values of the models were all greater than 0.9, indicating that the biomarkers had good diagnostic efficiency for PsO patients (**Figures 4A–C**). The results presented above indicated that the ANN models possessed exceptional forecasting proficiency for PsO. Based on the thresholds of *P* < 0.05, FDR < 0.25 and |NES| >1, GOT2, PYGL and SLC25A4 were significantly enriched in 70, 70 and 50 pathways, respectively. Commonly enriched pathways included the JAK-STAT signaling pathway and aminoacyl tRNA biosynthesis. (**Figures 4D–F**; **Supplementary Tables 7**-**9**). GGI network indicated that 20 genes were linked to the 3 biomarkers. These genes were primarily based on physical interactions (77.64%) and co-expression (8.01%), and these 20 genes were involved in related pathways, encompassing glycogen metabolic process, glucan metabolic process, and cellular glucan metabolic process, etc. (**Figure 4G**).

### 3.6 Biomarker-associated tissues, genes and diseases

Tissue localization, correlation, and disease prediction analyzes were conducted to comprehensively explore their roles and potential associations in PsO. Tissue localization analysis revealed that in the skin and related tissues, GOT2 and SLC25A4 show moderate expression, whereas PYGL shows low expression (**Supplementary Figure 2A**). Correlation analysis showed that SLC25A4 was significantly negatively correlated with PYGL (cor = -0.72, *P* < 0.001) and GOT2 (cor = -0.74, *P* < 0.001), respectively, while GOT2 and PYGL (cor = 0.78, *P* < 0.001) were significantly positively correlated (**Supplementary Figure 2B**). Disease prediction showed that SLC25A4, PYGL, and GOT were associated with necrosis, inflammation (**Supplementary Figure 2C**).

### 3.7 Immune cells and IFs of 3 biomarkers in PsO

**Figure 5:**
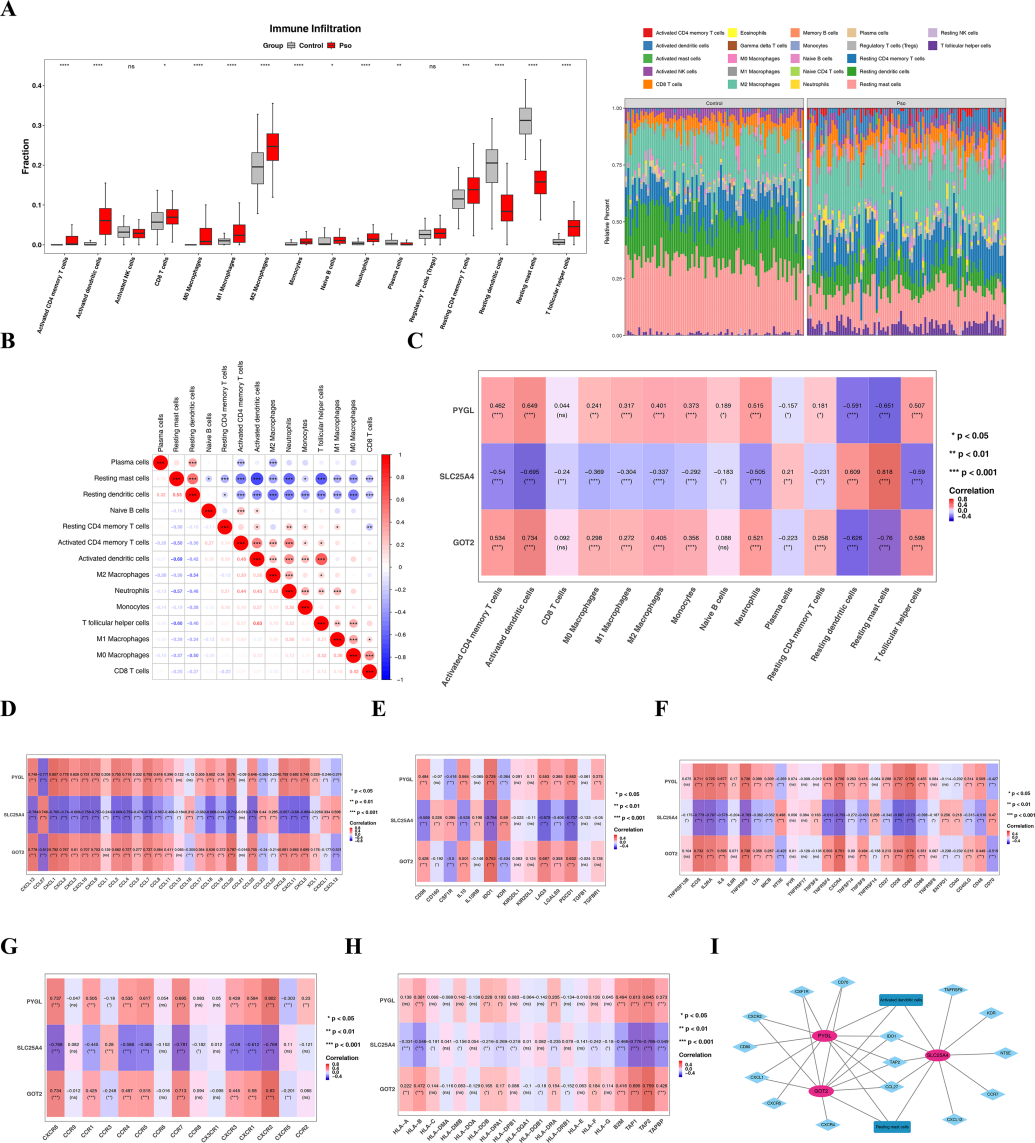
Immune infiltration analysis and correlation analysis of biomarkers with related immune factors. **(A)** Box plots of differential analysis of immune cell infiltration levels and stacked plots of relative proportions of immune cells. **(B)** Heatmap of correlation analysis between differential immune cells and differential immune cells. **(C)** Correlation plot between biomarkers and differential immune cells. Correlation analysis of biomarkers with related immune factors CRGs **(D)**, IIRGs **(E)**, ISRGs **(F)**, CRRGs **(G)**, MRGs **(H)**. **(I)** The interconnection network among biomarkers, differential immune cells, and immune factors. In the network, red represents genes, blue represents immune factors, and dark blue represents differential immune cells. *P < 0.05, **P < 0.01, ***P < 0.001, ****P < 0.0001.

Given the candidate genes’ association with inflammation and PsO’s link to the immune microenvironment, immune cell infiltration and IFs correlation analyzes were performed. Fourteen immune cells showed significant differences between PsO and control cohorts (*P* < 0.05) (**Figure 5A**). Plasma cells, resting dendritic cells (DCs) and resting mast cells were less infiltrated in PsO, while other cell types (except activated NK cells and regulatory T cells with no differences) were more infiltrated (*P* < 0.05). T follicular helper cells positively correlated with activated DCs (cor = 0.63, *P* < 0.001), and activated DCs negatively correlated with resting mast cells (cor = -0.69, *P* < 0.001) (**Figure 5B**). Resting mast cells positively correlated with SLC25A4 (cor=0.82, *P* < 0.001) but negatively with GOT2 (cor = -0.76, *P* < 0.001) (**Figure 5C**; **Supplementary Table 10**), indicating they may regulate PsO via resting mast cells.

Chemokine correlation analysis showed CXCL1 positively correlated with PYGL (cor=0.81, *P* < 0.001) and GOT2 (cor=0.79, P<0.001), while CCL27 negatively correlated with GOT2 (cor = -0.81, *P* < 0.001) and CXCL13 with SLC25A4 (cor = -0.78, *P* < 0.001) (**Figure 5D**; **Supplementary Table 11**). Immunosuppressants analysis revealed IDO1 positively correlated with GOT2 (cor = 0.78, *P* < 0.001) but negatively with SLC25A4 (cor = -0.76, *P* < 0.001) (**Figure 5E**; **Supplementary Table 12**).

Immunostimulatory factors, chemokine receptors and MHC analyzes showed CXCR4, CXCR2, TAP2 positively correlated with GOT2 (cor = 0.75, 0.83, 0.76, *P* < 0.001), while TNFRSF9, CCR7, TAP2 negatively correlated with SLC25A4 (cor = -0.78, -0.78, -0.79, *P* < 0.001) (**Figures 5F–H**; **Supplementary Tables 13**-**15**). This indicated GOT2 and SLC25A4 act synergistically and antagonistically with IFs, respectively. The IF-biomarker-DICs network showed 3 biomarkers interacted with 15 IFs and 2 DICs (**Figure 5I**), participating in PsO pathogenesis via these components.

### 3.8 Regulatory networks and drugs of 3 biomarkers in PsO

**Figure 6:**
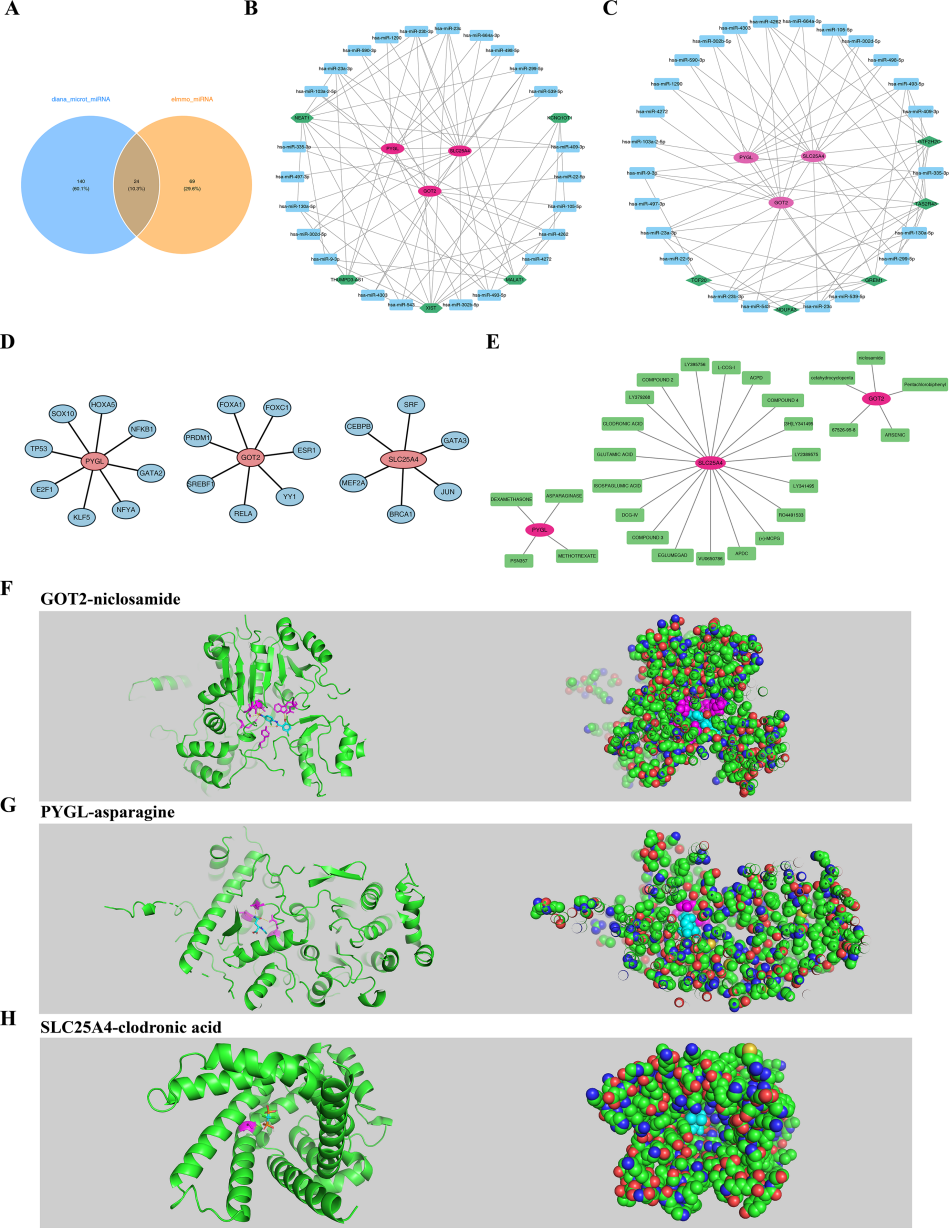
Construction of molecular regulatory network and molecular docking analysis. **(A)** Venn plot of intersection between miRNA and circRNA. **(B)** lncRNA - miRNA - mRNA network. In the figure, red represents genes, blue represents miRNAs, and green represents lncRNAs. **(C)** circRNA - miRNA - mRNA regulatory network. In the network, red represents genes, blue represents miRNAs, and green represents circRNAs. **(D)** TFs - mRNA regulatory network of biomarkers. Red represents biomarkers, and blue represents TFs. **(E)** Biomarker - drug association network. Red represents genes, and green represents drugs. Molecular docking results of GOT2 **(F)**, PYGL **(G)** and SLC25A4 **(H)**.

To investigate the role of the 3 biomarkers in PsO, non-coding RNA regulatory network analysis, TFs prediction, and drug-molecular docking analysis were conducted to comprehensively explore the upstream regulatory pathways and potential therapeutic targets of biomarkers. The results showed that 24 key miRNAs (such as hsa-miR-23a-3p, hsa-miR-103a-2-5p, and hsa-miR-1290 etc.)were selected via 3 biomarkers (**Figure 6A**; **Supplementary Table 16**). Total of 5 lncRNAs (NEAT1, KCNQ1OT1, THUMPD3-AS1, MALAT1 and XIST) and 5 cirRNAs (GTF2H2C, TAS2R43, TCF20, GREM1 and NDUFA3) were predicted via these 11 key miRNAs. (**Figures 6B, C**; **Supplementary Tables 17**, **S18**). 21 TFs (NFKB1, GATA, PRDM1, FOXA1, GATA3 and JUN etc.)were predicted based on 3 biomarkers (**Figure 6D**). 28 drugs or molecular compounds associated with the 3 biomarkers were predicted by DSigDB and DGIdb, including asparagine, methotrexate, and dexamethasone (associated with PYGL), clodronic acid, eglumegad, glutamic acid and DCG-IV (associated with SLC25A4), and niclosamide, pentachlorobiphenyl, and octahydrocyclopenta (associated with GOT2) (**Figure 6E**; **Supplementary Table 19**). Molecular docking results showed that GOT2, PYGL and SLC25A4 displayed a strong binding affinity for niclosamide, asparagine and clodronic acid, respectively exhibiting a binding free energy of -8.9 kcal/mol (**Table 3**; **Figures 6F–H**).

**Table 3:** Binding energies of biomarkers to key drug or molecular compounds.

Gene	Drug	Binding energies(kcal/mol)
GOT2	niclosamide	-8.9
PYGL	asparagine	-5.0
SLC25A4	clodronic acid	-5.4

### 3.9 Identification of keratinocytes and KC subtypes in PsO

**Figure 7:**
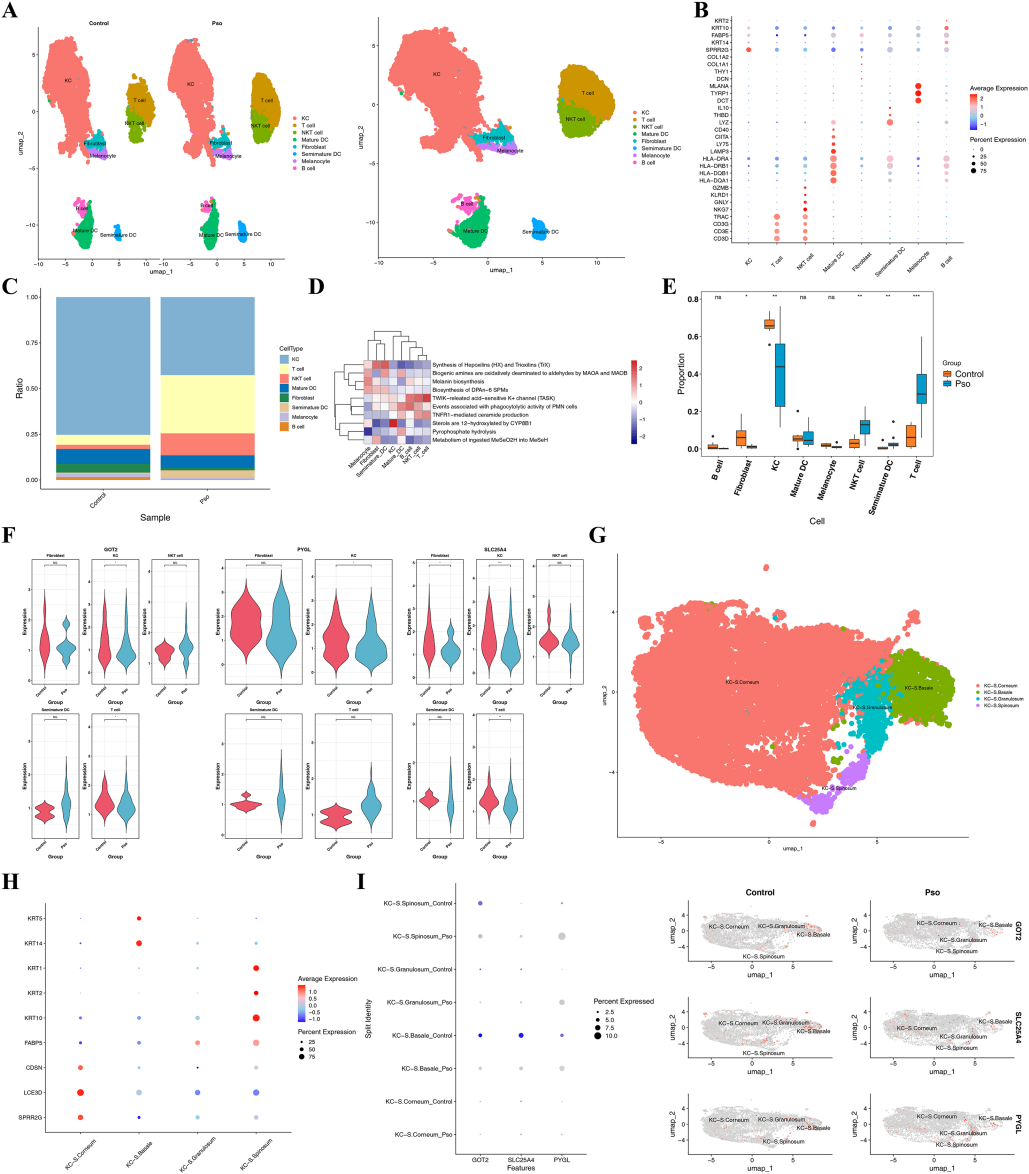
Cell clustering and annotation analysis. **(A)** UMAP plot of the clustered cell clusters after annotation. **(B)** Bubble plot of the expression of marker genes in different cells. **(C)** Bar chart of cell proportions. **(D)** Enrichment analysis of cell clusters. **(E)** Box plot of cell proportions, where ns represents insignificant, *P < 0.05, **P < 0.01, ***P < 0.001. **(F)** Violin plot of the expression of biomarkers GOT2, PYGL and SLC25A4. **(G)** UMAP plot of re-annotation of subtype marker genes of KC cells. **(H)** Bubble plot of the expression of marker genes in different cells after re-annotation. **(I)** Expression of three biomarkers GOT2, SLC25A4 and PYGL in PsO patients.

To clarify the cell-specific distribution and functional targets of core biomarkers in PsO, cell subset typing and biomarker expression localization analyzes were conducted. Following quality control filtration of the GSE220116 dataset (retaining 26,225 of 35,245 cells and 23,313 genes), 2,000 HVGs were identified, and 19 cell clusters were obtained by clustering analysis of the top 30 PCs (**Supplementary Figures 3A–F**). As shown in **Figure 7A**, 19 cell clusters were divided into 8 different cell types, and these cell types consisted of KC, T cells, natural killer (NK) T cells (NKT cells), mature DC, fibroblasts, semimature DC, melanocytes, and B cells. The expression levels of marker genes in 8 separate cell types were demonstrated by the bubble chart shown in **Figure 7B**. The bar graph displayed that the proportion of KC cells was significantly reduced in PsO compared to the control group. (**Figure 7C**). The functional analysis indicated that the sterols are 12-hydroxylated by CYP8B1 was activated in KC (**Figure 7D**). The Wilcoxon test showed that KC abundance was significantly lower in PsO compared to controls (*P* < 0.05, **Figure 7E**). Furthermore, it revealed that differential expression of key genes: GOT2 in T cells and KC, PYGL in KCs, and SLC25A4 in KCs, T cells, and fibroblasts (*P* < 0.05, **Figure 7F**). Thus, KC were designated as key cells and re-annotated into 4 KC subtypes, namely KC-S.Corneum, KC-S.Basale, KC-S.Granulosum, and KC-S.Spinosum (**Figure 7G**). The bubble chart showed expression of subtype-marker genes in 4 KC subtype (**Figure 7H**). The three biomarkers GOT2, SLC25A4, and PYGL, particularly SLC25A4, exhibited significantly lower expression in PsO compared to the control (**Figure 7I**).

### 3.10 Function, cell cycle and cellular communication of KC in PsO

**Figure 8:**
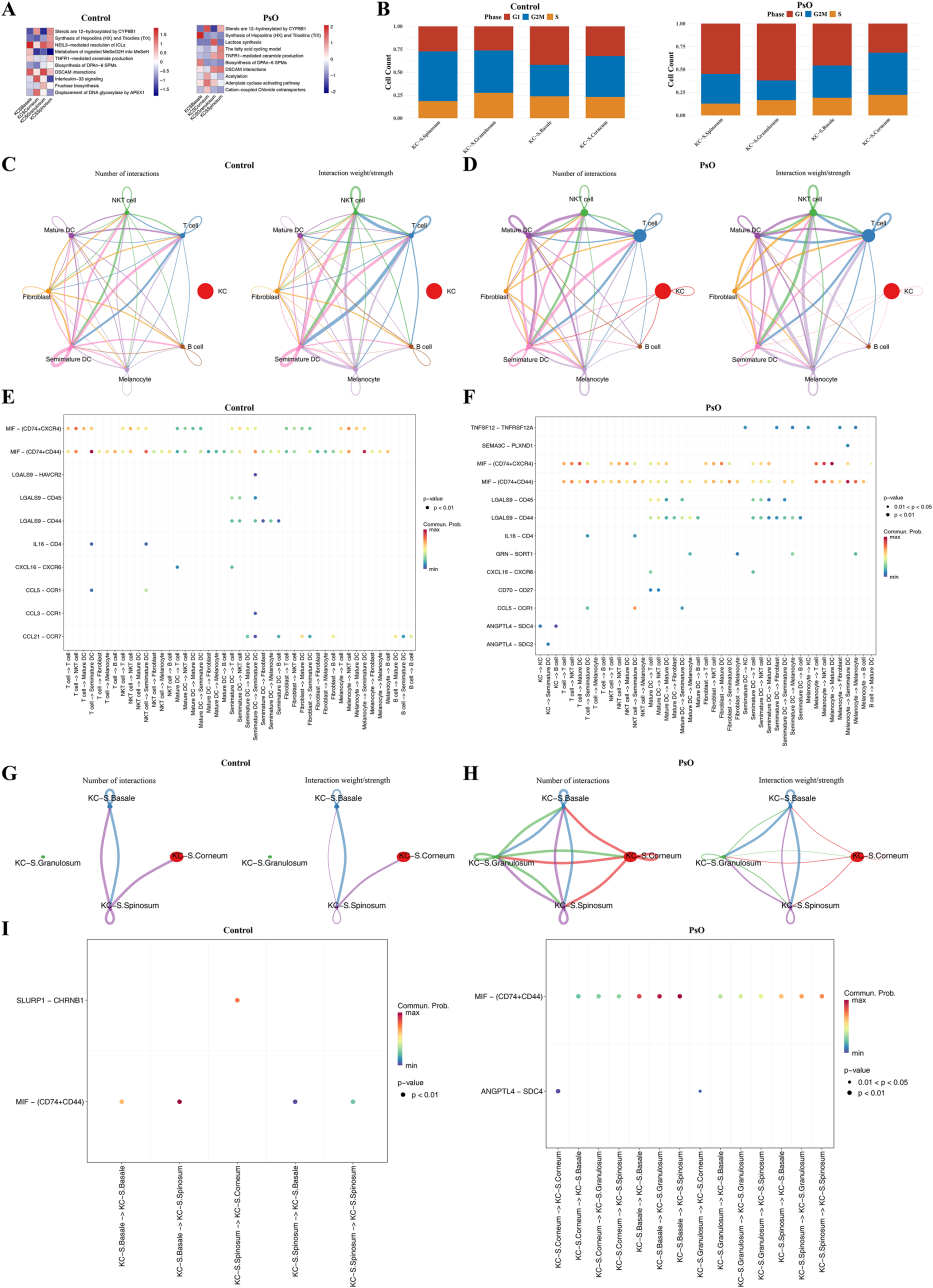
Cell communication analysis. **(A)** Functional enrichment analysis of KCs in the control group and the PsO group. **(B)** Cell cycle analysis of the control group and the PsO group. Number and weight diagrams of cell communication analysis in the control group **(C)** and the PsO group **(D)**. Probability of ligand-receptor interaction between cells in the control group **(E)** and the PsO group **(F)**. Number and weight diagrams of interactions between cell subsets in different samples of the control group **(G)** and the PsO group **(H)**. **(I)** Probability of ligand-receptor interaction between cells in the control group and the PsO group.

To further analyze the role of KCs in PsO, we performed a functional analysis of these cells. The results revealed significant differences in the functional enrichment pathways of different KCs between the control and PsO. Specifically, the synthesis of hepoxilins (HX) and trioxilins (TrX) in KC-S.basal was activated in PsO compared to controls. (**Figure 8A**). Cell cycle analysis showed that in controls, KC-S.Spinosum and KC-S.Granulosum were predominantly in the G2M phase, indicating that these cells were in active cell division. In PsO samples, KC-S.Spinosum and KC-S.Granulosum cells were predominantly in the G1 phase (the preparatory period for cell division) compared to control, with a corresponding significant decrease in the S and G2/M phases, indicating a weakened proliferative ability of these cells (**Figure 8B**).

The cell communication analysis findings suggested that in control samples, the interactions between T cells and T cells, T cells and NKT cells, T cells and semimature DC, as well as NKT cells, mature DC and semimature DC were more conspicuous. Remarkably, T cells communicated more intensively with T cells, NKT cells, mature DC and semimature DC were more intense (**Figure 8C**). Conversely, in the PsO samples, there were more frequent interactions between KC and KC, KC and B cells, as well as KC and semimature DC. Intriguingly, the associations between KC and KC, KC and B cells, as well as KC and semimature DC were more intense (**Figure 8D**). The molecular results of intercellular communication signals showed that in control samples, the primary signals sent from T cells to semimature DC were MIF-(CD74+CD44) (**Figure 8E**). Whereas in PsO samples, the primary signals sent from melanocytes to semimature DC were MIF-(CD74+CXCR4) (**Figure 8F**). Furthermore, cell–cell communication analysis revealed distinct interaction patterns among KC subtypes in control and PsO groups. In control samples, all four KC subtypes formed a dense and relatively balanced interaction network, with prominent connections among KC-S.Basale, KC-S.Spinosum and KC-S.Corneum, supporting epidermal structural and functional homeostasis (**Figure 8G**). In PsO samples, the network became more polarized, with KC-S.Basale and KC-S.Spinosum forming the dominant communication axis, whereas KC-S.Granulosum showed only weak or absent interactions (**Figure 8H**). Notably, MIF–(CD74+CD44) was identified as the primary intercellular communication signal from KC-S.Basale to KC-S.Spinosum in both PsO and control samples (**Figure 8I**). This shift may enhance KC-S.Basale regulatory roles, contributing to pathological epidermal proliferation and differentiation.

### 3.11 Pseudotime trajectory of KC in PsO

**Figure 9:**
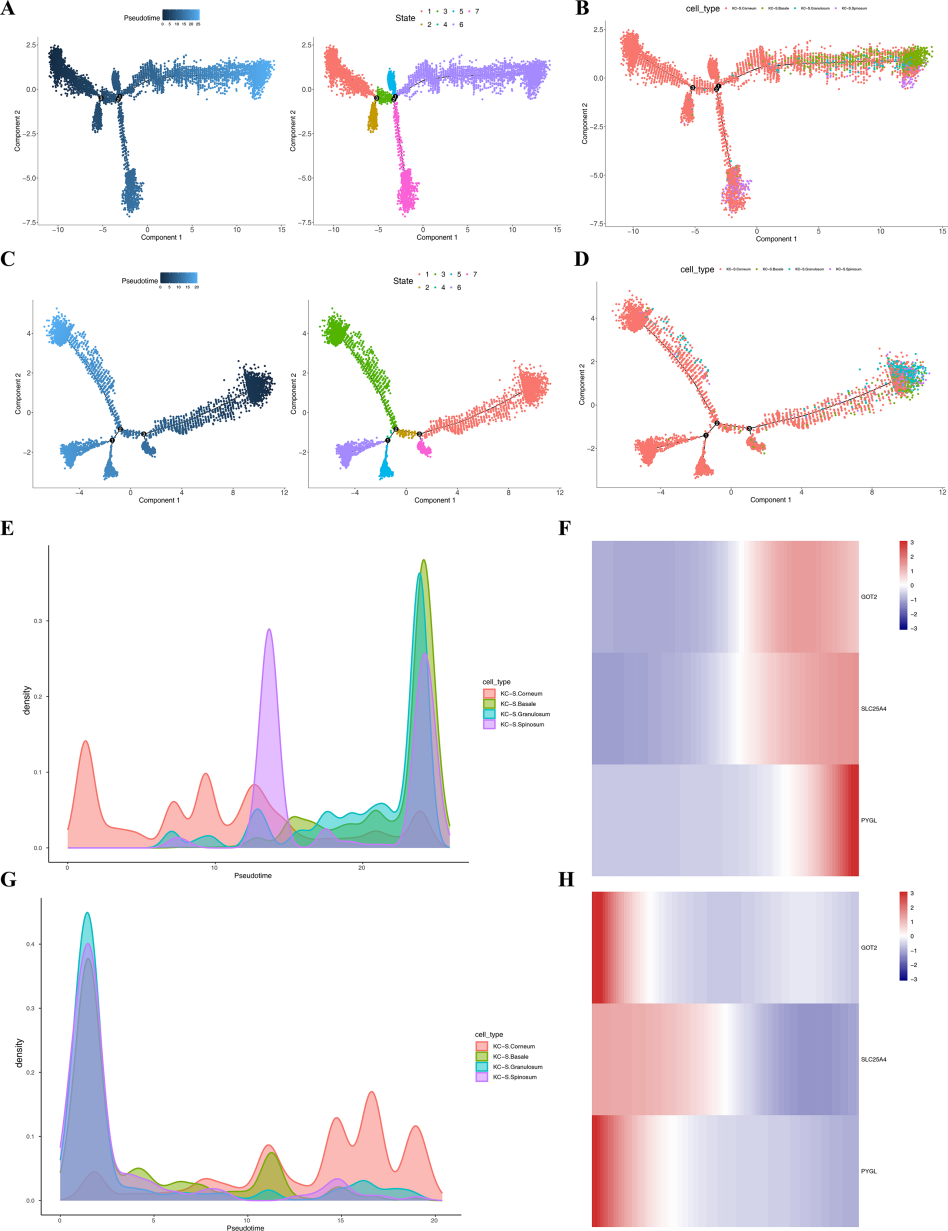
Pseudotemporal analysis. **(A)** Results of pseudotemporal analysis plots of different cell State maps and cell types in the control group. **(B)** Pseudotemporal analysis plots of different cell types in the control group. **(C)** Results of pseudotemporal analysis plots of different cell State maps and cell types in the disease group. **(D)** Pseudotemporal analysis plots of different cell types in the disease group. **(E)** Pseudotemporal density plot of KC cells in the control group. **(F)** Pseudotemporal results of genes in the control group. **(G)** Pseudotemporal density plot of KC cells in the disease group. **(H)** Pseudotemporal results of genes in the disease group.

To determine the developmental stages affected by the core biomarkers, we employed pseudotime analysis. This revealed that KC cells were observed to differentiate from left to right, ultimately forming 7 distinct states in the control samples (**Figure 9A**). KC-S.Granulosum and KC-S.Basale were predominantly in the terminal stage of KC differentiation (**Figure 9B**). In contrast, within PsO samples, KC differentiation proceeded from right to left, also forming 7 distinct states (**Figure 9C**). The trajectory plot showed that KC-S.Granulosum and KC-S.Basale were mainly in the early stage of KC differentiation in PsO (**Figure 9D**). Meanwhile, in control samples, high peaks were present in KC-S.Corneum during the early stage, while a peak of KC-S.Spinosum appeared in the mid-stage. In the late stage, peaks of KC-S.Basale, KC-S.Granulosum, and KC-S.Spinosum emerged (**Figure 9E**). PYGL was mainly expressed in the late stages, while GOT2 and SLC25A4 were mainly expressed in the middle and late stage of KC development (**Figure 9F**). In the PsO samples, high peaks were present in KC-S.Basale, KC-S.Spinosum, and KC-S.Granulosum during the early stage, while a peak of KC-S.Corneum and KC-S.Basale appeared in the mid-stage. In the late stage of KC development, peaks of KC-S.Corneum emerged (**Figure 9G**). SLC25A4 was mainly expressed in the early and middle stages, while GOT2 and PYGL were mainly expressed in the early stage of KC development (**Figure 9H**). These results suggested that PYGL, GOT2, and SLC25A4 might influence keratinocyte state transitions along the pseudotime trajectory and play crucial roles in PsO.

### 3.12 Subclustering analysis of T cells and fibroblasts in PsO

**Figure 10:**
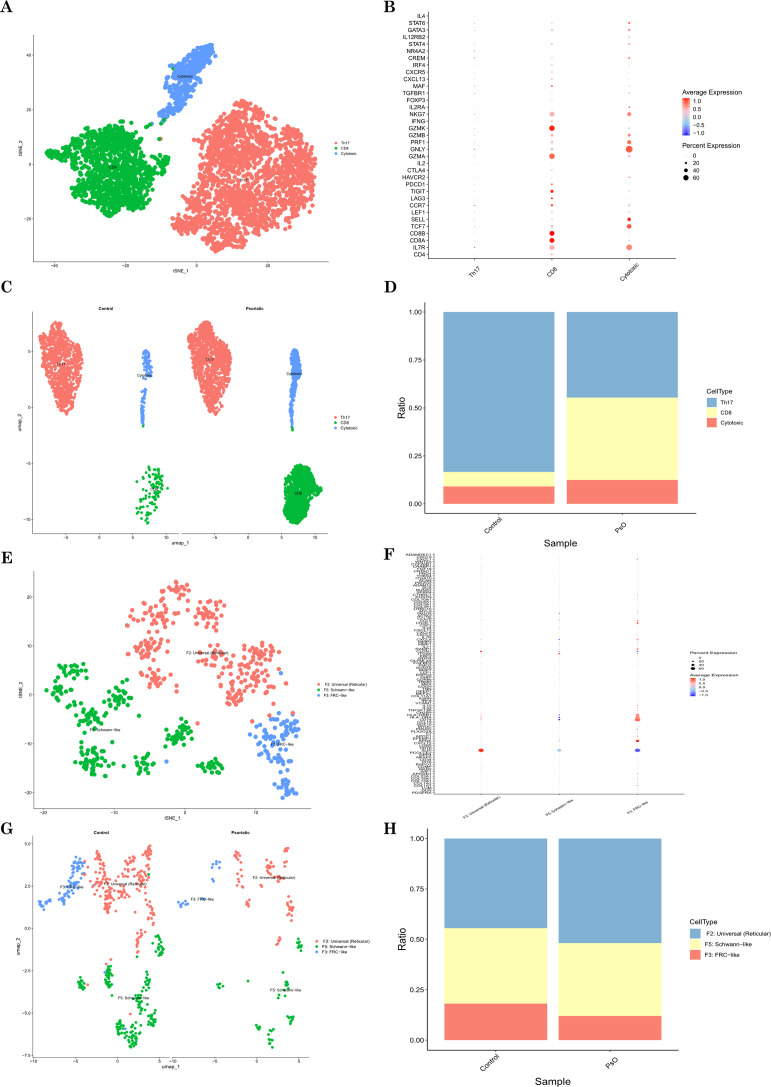
Subclustering analysis of T cells and fibroblasts in PsO. **(A)** tSNE plot of annotated T cell subtypes (Th17, CD8^+^ T cells, Cytotoxic T cells). **(B)** Bubble plot of marker gene expression across annotated T cell subtypes. **(C)** UMAP plot showing T cell subtype distribution in control and PsO samples separately. **(D)** Bar chart of T cell subtype proportions in control and PsO groups. **(E)** tSNE plot of annotated fibroblast subtypes (F2: Universal Reticular, F5: Schwann-like, F3: FRC-like). **(F)** Bubble plot of marker gene expression across annotated fibroblast subtypes. **(G)** UMAP plot showing fibroblast subtype distribution in control and PsO samples separately. **(H)** Bar chart of fibroblast subtype proportions in control and PsO groups.

To further characterize the immune and stromal microenvironments in PsO, T cells and fibroblasts were independently extracted and subjected to subclustering analyzes. Quality control, PC selection, and optimal resolution determination for both cell populations are provided in **Supplementary Figures 4** and **S5**. For T cell subclustering, three subtypes were identified and annotated as Th17, CD8^+^ T cells, and Cytotoxic T cells (**Figures 10A, B**). Th17 cells were characterized by IRF4, CREM, and NR4A2; CD8^+^ T cells by CD8A, CD8B, GZMA, and GZMK with co-expression of exhaustion markers TIGIT and LAG3; and Cytotoxic T cells by GNLY, PRF1, NKG7, alongside naive markers TCF7 and SELL. Although Th17 cells were predominant in both groups, their proportion was notably reduced in PsO while CD8^+^ T cells markedly expanded, suggesting a shift from Th17 dominance toward cytotoxic infiltration in psoriatic lesions (**Figures 10C, D**). For fibroblast subclustering, three subtypes were annotated as F2: Universal (Reticular) fibroblasts (PI16, CD34, SLPI), F5: Schwann-like fibroblasts (SCN7A, NGFR, CLDN1), and F3: FRC-like fibroblasts (HLA-DRA, CD74, APOE, EFEMP1) (**Figures 10E, F**). UMAP visualization confirmed distinct spatial distributions between groups (**Figure 10G**). F2 Universal fibroblasts were most abundant and further expanded in PsO, while F3 FRC-like fibroblasts were relatively reduced, and F5 Schwann-like fibroblasts showed modest increase (**Figure 10H**). The reduction of immunoregulatory F3 FRC-like fibroblasts alongside F2 expansion may reflect stromal remodeling that compromises immune homeostasis in psoriatic lesions.

### 3.13 Experimental verification

**Figure 11:**
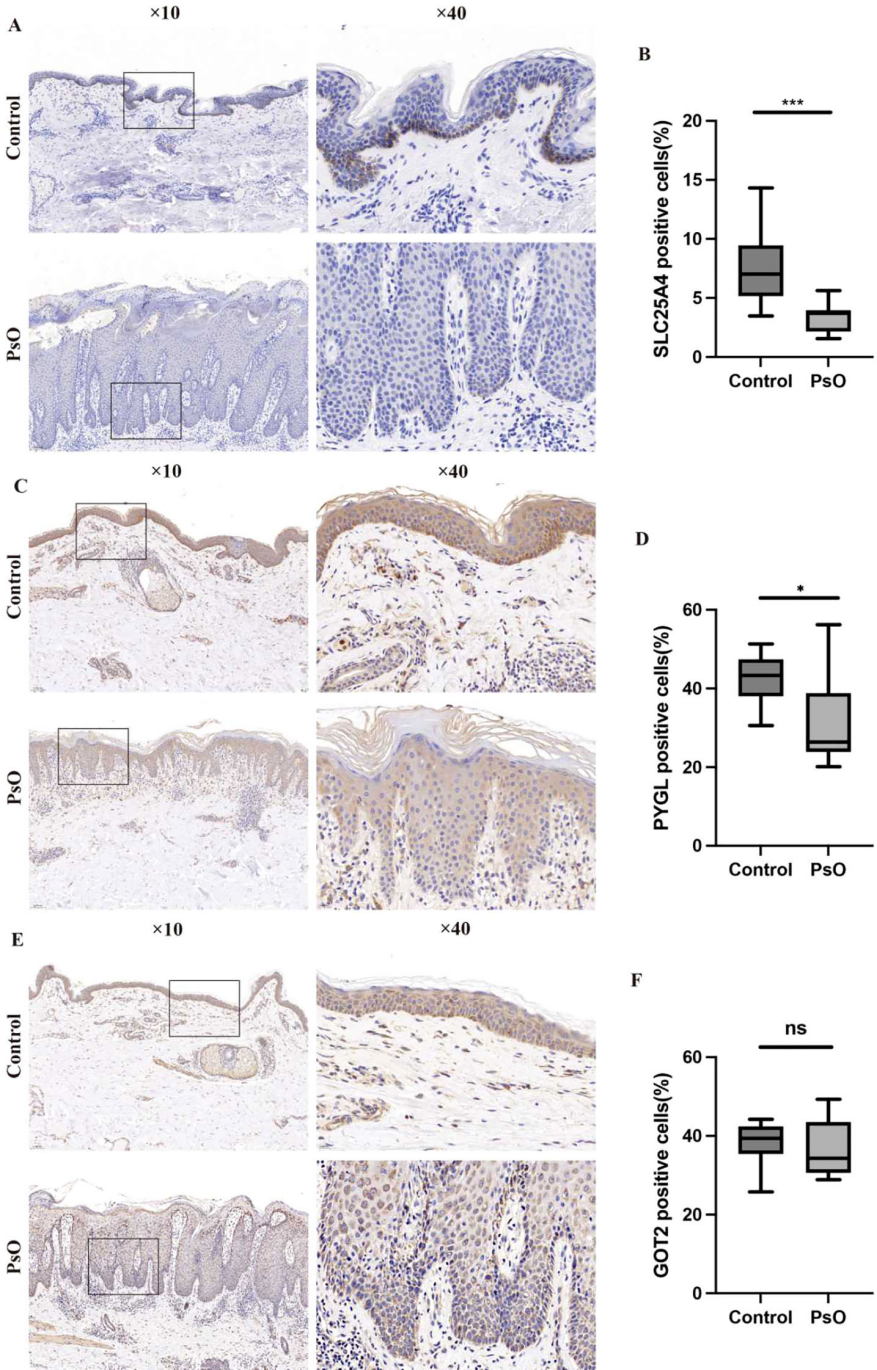
Immunohistochemical analysis of biomarkers. **(A, B)** Validation of protein expression by IHC analysis of the candidate biomarker SLC25A4. **(C, D)** Validation of protein expression by IHC analysis of the candidate biomarker PYGL. **(E, F)** Validation of protein expression by IHC analysis of the candidate biomarker GOT2, ns represents insignificant, n = 10 per group, *P < 0.05, ***P < 0.001 vs control.

To further validate the roles of SLC25A4, PYGL, and GOT2 in PsO, we performed IHC analysis on tissue samples from 10 PsO patients and 10 healthy controls. Quantitative results showed significantly reduced expression of SLC25A4 (*P* < 0.001) and PYGL (*P* < 0.05) in PsO compared to controls, consistent with our transcriptional data, while GOT2 expression showed no significant difference (*P*>0.05) (**Figure 11**). The expression changes of SLC25A4 was consistent with the previous transcriptional-level findings. Therefore, we speculated that SLC25A4 may play a significant role in PsO.

**Figure 12:**
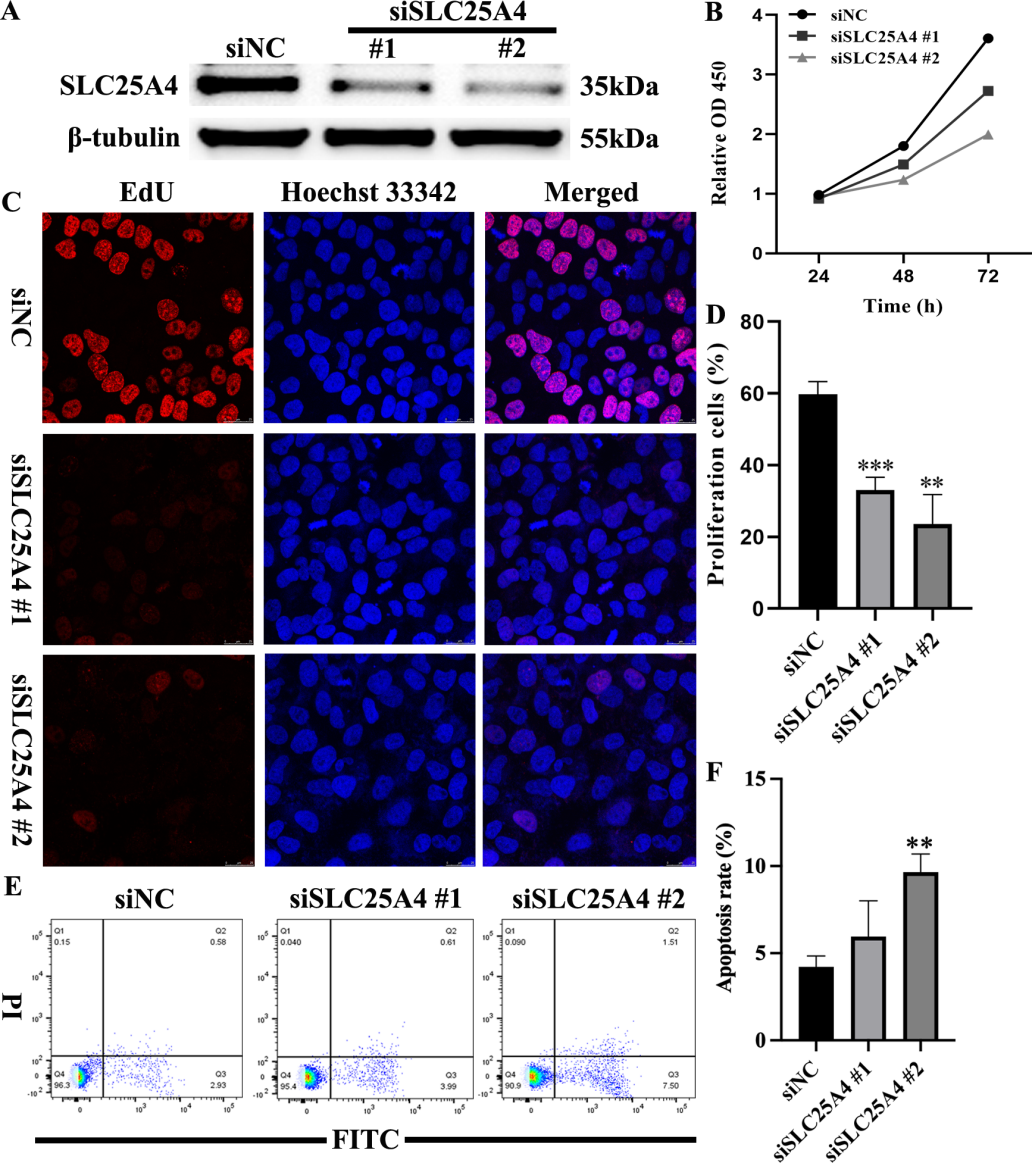
Inhibition of SLC25A4 impedes cell proliferation in HaCaT cells. **(A)** The protein level of SLC25A4 in HaCaT cells with silencing SLC25A4. **(B)** Cell viability for HaCaT cells with silencing SLC25A4. **(C, D)** Cell proliferation of HaCaT cells with silencing SLC25A4 measured by EdU incorporation assay. **(E, F)** Cell apoptosis of HaCaT cells with silencing SLC25A4 determined by flow cytometry. Data are shown as mean ± SD. *P* values were calculated using student *t* test. **P < 0.01, ***P < 0.001vs siNC.

To further investigate the implications of the above findings, we knocked down SLC25A4 *in vitro* to elucidate its potential physiological roles in PsO. Two siRNAs specifically targeting SLC25A4 exhibited obvious inhibitory effect (**Figure 12A**). A significant reduction in the cell viability of HaCaT cells upon the silencing of SLC25A4 (**Figure 12B**). The EdU-positive cells were significantly decreased after silencing SLC25A4 (**Figures 12C, D**, *P* < 0.01). Meanwhile, SLC25A4 knockdown significantly increased the apoptotic rate of HaCaT cells (**Figures 12E, F**, *P* < 0.01). These data suggest a mechanism whereby SLC25A4 downregulation in PsO contributes to the pathogenesis mainly through the inhibition of cell proliferation and the induction of apoptosis.

## 4 Discussion

In this study, we integrated WGCNA, differential expression analysis, Venn intersection, MR, and multiple machine-learning algorithms to identify three lactate metabolism-related biomarkers (GOT2, PYGL, and SLC25A4) in PsO. These genes achieved high diagnostic accuracy. They are enriched in pathways such as JAK–STAT signaling and aminoacyl-tRNA biosynthesis and correlate with key immune cells (e.g., resting mast cells, T follicular helper cells), and mediators (CXCL1, IDO1, TNFRSF9). Single-cell analysis showed that KCs were identified as the key cell population. In addition, they show strong predicted interactions with niclosamide (GOT2), asparagine (PYGL) and clodronic acid (SLC25A4) and may influence early keratinocyte differentiation. *In vitro* experiments demonstrated that downregulating the SLC25A4 gene inhibited cell proliferation and promoted apoptosis. Together, these findings establish a link between lactate metabolism and PsO, offering potential biomarkers and therapeutic entry points for personalized PsO management.

Solute carrier family 25 member 4 (SLC25A4), also known as adenine nucleotide translocase 1 (ANT1), is an ADP/ATP transporter located in the inner mitochondrial membrane and essential for maintaining oxidative phosphorylation and mitochondrial integrity (60). Beyond its role in energy metabolism, emerging evidence indicates that SLC25A4 is involved in inflammatory regulation: its loss in idiopathic pulmonary fibrosis can lead to mitochondrial dysfunction and chronic inflammation (61), and its downregulation during SARS-CoV-2 infection is associated with impaired oxidative phosphorylation and elevated cytokine production (62). Mechanistically, SLC25A4 deficiency may promote reactive oxygen species (ROS) accumulation and activate the NLRP3 inflammasome (63). Here, SLC25A4 demonstrated the most robust cross-platform consistency across transcriptomic, single-cell, and IHC analyzes. Transcriptomic, single-cell, and IHC analyzes collectively demonstrated that SLC25A4 expression was persistently lower in PsO lesions than in controls, with particularly pronounced downregulation in basal-layer KCs. The IHC confirmation at the protein level is critical because it localizes the defect to the main proliferative compartment of the epidermis—the basal keratinocytes. Basal keratinocytes constitute the main proliferative compartment that sustains epidermal renewal and psoriatic hyperplasia, and their metabolic status is tightly coupled to mitochondrial function and ATP supply (64, 65). Functionally, SLC25A4 knockdown in HaCaT cells (a basal-like KC line) markedly reduced cell viability and proliferation while increasing apoptosis, suggesting that SLC25A4 deficiency may skew keratinocytes toward diminished proliferation and enhanced cell death. These results suggest that PsO lesions are not merely characterized by excessive proliferation, but are also accompanied by an imbalance in the homeostasis between proliferation and apoptosis. This protein-level evidence also aligns with the protective causal direction suggested by our MR analysis, supporting the interpretation that SLC25A4 loss may actively contribute to disrupted proliferation–apoptosis balance in keratinocytes. Linking back to our bioinformatics findings, the WGCNA module containing SLC25A4 was strongly correlated with “proliferation-apoptosis” pathways, and single-cell trajectory analysis placed SLC25A4 expression at an early KC differentiation stage in PsO, whereas in controls it appeared later. Thus, IHC not only validated the transcriptomic trend but also pinpointed the basal KC as the cellular origin of SLC25A4-related metabolic vulnerability, mechanistically connecting reduced mitochondrial ADP/ATP transport to premature differentiation and disrupted proliferation-apoptosis homeostasis observed in our pseudotime and cell-cycle analyzes. Previous studies support this notion. For example, downregulation of GSDMB in psoriatic lesions suppresses HaCaT cell proliferation and promotes apoptosis (66); the pro-apoptotic protein p53 is upregulated, whereas the anti-apoptotic protein Bcl-2 is downregulated (67). Targeting molecules such as PRDX2, BRD4, or RSAD2 can likewise inhibit keratinocyte proliferation and induce apoptosis (68, 69). Taken together, the SLC25A4 deficiency identified in our study may represent a metabolic manifestation of disrupted “proliferation–apoptosis homeostasis” in PsO.

Liver glycogen phosphorylase (PYGL) is a key enzyme in glycogenolysis and contributes to the generation of substrates for glycolysis (70, 71). Here, PYGL exhibited a striking transcript-protein discordance. While mRNA levels of PYGL were significantly upregulated in PsO lesions by transcriptomic analysis (AUC > 0.9), IHC revealed a marked decrease in PYGL protein abundance in PsO epidermis compared to normal skin. This opposite trend, consistent with Martinez-Navarro et al. (72), suggesting that PYGL may be finely regulated by the inflammatory microenvironment at post-transcriptional or translational levels. By integrating this IHC finding with our regulatory network analysis (which predicted NEAT1/miR-130a-5p and transcription factors such as NFKB1 as upstream regulators of PYGL), we now hypothesize that PsO-associated inflammatory cytokines (e.g., TNF-α, IL-17) may activate NFKB1, which in turn could upregulate specific microRNAs or RNA-binding proteins that suppress PYGL translation. The IHC evidence thus transforms PYGL from a mere diagnostic transcript marker into a model system for studying how inflammation decouples mRNA abundance from protein function in glycogen/lactate metabolism—a finding that would have been impossible using transcriptomics alone.

Glutamic-oxaloacetic transaminase 2 (GOT2), the mitochondrial isoform of aspartate aminotransferase, is essential for amino acid metabolism and the TCA cycle, catalyzing the reversible conversion of glutamate and oxaloacetate to α-ketoglutarate and aspartate and participating in the malate-aspartate shuttle to maintain NADH/redox homeostasis (73–75). There were few literatures on the relationship between GOT2 and PsO. Here, GOT2 presented a different pattern: mRNA was consistently elevated in PsO lesions (AUC > 0.9), but IHC showed no significant difference in total protein levels between PsO and normal epidermis. This dissociation led us to re-examine our bioinformatics data. Pathway enrichment indicated that GOT2 participates in aminoacyl-tRNA biosynthesis and the malate-aspartate shuttle, but these functions depend on enzyme activity and subcellular localization rather than total protein abundance. We therefore propose that GOT2 in PsO may be regulated post-translationally (e.g., by acetylation, phosphorylation, or interaction with partner proteins) or may exhibit altered mitochondrial vs. cytosolic partitioning—mechanisms that would not be detected by conventional IHC measuring total protein. Supporting this, our single-cell data showed that GOT2 expression was enriched in early-differentiating KCs, and its correlation with IDO1 and CXCR2 suggests context-dependent metabolic-immune crosstalk. The IHC finding, rather than contradicting the transcriptomic data, redirects attention to post-translational and compartment-specific regulation. Future studies using activity-based assays or subcellular fractionation will be required to test whether GOT2 enzymatic function, rather than its protein level, drives amino acid flux and redox balance in psoriatic KCs. This suggests that GOT2 may function in PsO through post-transcriptional or context-dependent mechanisms, influencing amino acid metabolism and mitochondrial homeostasis rather than through changes in its overall abundance. Taken together, IHC validation confirmed the mRNA–protein discordance for both PYGL and GOT2 at the tissue level, reinforcing the notion that transcriptional upregulation does not necessarily translate to increased protein abundance in the psoriatic microenvironment, and highlighting that post-transcriptional or post-translational regulatory mechanisms may be operative for both genes. These findings underscore the importance of integrating multi-level evidence when interpreting transcriptomic biomarkers in inflammatory disease contexts.

Beyond their direct involvement in lactate and energy metabolism, the three biomarkers (GOT2, PYGL, and SLC25A4) converge on several signaling routes central to Psoriatic inflammation. Pathway enrichment highlighted common participation in JAK–STAT signaling. The JAK–STAT pathway is a central signaling axis for many cytokines; through phosphorylation cascades it regulates gene transcription and plays a key role in cell proliferation, differentiation, and immune inflammation (76, 77). In PsO, this pathway is persistently activated by cytokines such as IL-23 and IFN-γ, and especially the aberrant activation of STAT3 can directly drive KCs hyperproliferation and shape the inflammatory microenvironment, representing a well-recognized core pathogenic mechanism (78, 79). Accordingly, small-molecule inhibitors targeting JAK–STAT (e.g., tofacitinib, upadacitinib) have become important therapeutic options for moderate-to-severe PsO (80, 81). KCs express abundant cytokine receptors and finely sense external stimuli via the JAK–STAT pathway; for example, IL-4 induces KCs to produce CCL26 through the JAK1/2–STAT6 axis, and aberrant expression of keratin 6A can intrinsically activate JAK1–STAT3 signaling (82, 83), indicating that the intrinsic JAK–STAT status of KCs is crucial for skin homeostasis and inflammation. Previous work has shown that SLC25A4 (also known as ANT1) interacts with the phosphatase SHP2 to prevent mitochondrial membrane potential collapse and excessive ROS release, thus limiting NLRP3 inflammasome activation (63). Taken together, we speculate that dysregulated SLC25A4 expression not only directly disrupts the balance between energy metabolism and survival in KCs, but that the resulting metabolic stress may further perturb JAK–STAT signaling networks within KCs, thereby cooperating with classical inflammatory axes to drive aberrant KC differentiation and persistent inflammation.

CIBERSORT-based deconvolution revealed broad remodeling of the immune landscape in PsO. Plasma cells, resting dendritic cells and resting mast cells were reduced, whereas several activated immune subsets were increased, consistent with a shift toward a highly inflammatory milieu. The strong positive correlation between T follicular helper cells and activated DCs, together with experimental evidence that keratinocyte polyamine excess and cGAS–STING activation in DCs promote PsOriasiform inflammation (84, 85), supports a DC–T-cell amplification loop in PsO lesion. CXCL1 showed strong positive correlations with both PYGL and GOT2, and GOT2 was also correlated with CXCR2/CXCR4. By contrast, SLC25A4 was negatively correlated with molecules such as TNFRSF9 and TAP2, whereas GOT2 was positively correlated with IDO1 and TAP2. Given that CXCL1 upregulation exacerbates psoriasiform dermatitis (86) and that IDO1 deficiency promotes inflammation (87), these findings suggest that PYGL and GOT2 may facilitate neutrophil recruitment and epidermal inflammation via the CXCL1–CXCR2 axis. Overall, GOT2 may amplify inflammation by cooperating with chemokine signaling and antigen-presentation pathways. SLC25A4 appears to be associated with a relatively immunosuppressive state; and PYGL may link glycogen/lactate metabolism to chemokine-driven immune-cell recruitment.

Our regulatory network analysis indicates that GOT2, PYGL, and SLC25A4 are embedded in a multilayered regulatory network. Among these, lncRNA NEAT1 may regulate SLC25A4 by sponging miR-130a-5p/miR-23a-3p, which is consistent with the known role of NEAT1 in modulating keratinocyte inflammation in PsO (49, 88). The predicted transcription factors (such as NFKB1, GATA2, PRDM1, and GATA3) also correspond to PsO-related signaling axes (89, 90). Molecular docking further suggests that niclosamide, asparagine, and clodronic acid may respectively target these three genes, and niclosamide has already been shown to possess anti-psoriatic activity (91). Taken together, these findings imply that these three lactate metabolism–related genes may be coordinately regulated by upstream non-coding RNAs and transcription factors, and may serve as potential targets of small-molecule drugs, thereby jointly influencing the course of PsO.

KCs are now recognized as central drivers rather than passive targets in PsO, with their hyperproliferation, abnormal differentiation and crosstalk with immune cells being crucial for lesion formation (64, 65). Our single-cell analysis showed that, although the overall proportion of KCs in PsO was significantly lower than in control, they still constitute the core cellular population of the epidermis. We further systematically delineated four functionally heterogeneous KC subtypes in PsO (KC-S.Basale, KC-S.Spinosum, KC-S.Granulosum, and KC-S.Corneum), consistent with the degree of epithelial heterogeneity reported in recent scRNA-seq studies (24). This finding suggests that KC dysfunction may arise not only from quantitative changes, but more importantly from profound reshaping of their internal subgroup composition and functional states. Meanwhile, we also found that the biosynthetic pathways of the pro-inflammatory lipid mediators hepoxilins (HX) and trioxilins (TrX) were selectively activated in the KC-S.Basale subtype. This is consistent with earlier reports of elevated HX/TrX levels in PsO lesions (92), thereby linking dysregulated lipid metabolism to the pathogenic effects of specific KC subpopulations. Although the classical view holds that psoriatic KCs are highly proliferative and resistant to apoptosis (93), our cell-cycle analysis showed a shift in the cell-cycle distribution of KC-S.Spinosum and KC-S.Granulosum in PsO from the S/G2M phases toward the G1 phase, indicating a relative reduction in actively proliferating cells in the suprabasal regions. This is consistent with recent single-cell studies suggesting the presence of heterogeneous KC subpopulations in PsO (24). Notably, we speculate that SLC25A4, which is markedly downregulated in basal KCs in PsO, may be a key regulator of this altered proliferative state and the cell-cycle arrest phenotype.

Pseudotime trajectory analysis further revealed that basal and granular-layer keratinocytes (KC-S.Basale and KC-S.Granulosum) in PsO enter the differentiation state earlier than those in control. Moreover, PYGL, GOT2, and SLC25A4 are mainly expressed at early stages of KC differentiation in PsO, whereas they are expressed at later stages in controls, indicating that metabolic reprogramming is an early event in KC abnormalities. At the same time, cell–cell communication analysis showed enhanced interactions among KCs and between KCs and immune cells in PsO, with the MIF–(CD74+CD44) signaling axis being particularly critical, consistent with reports that this pathway can drive KC proliferation and PsO-like dermatitis (94). Together with the known regulatory effects of IL-17 and related signals on KCs (93, 95, 96), we propose that metabolically reprogrammed basal KCs act as a hub that integrates inflammatory signals, lipid mediators, and intercellular communication, thereby reshaping KC differentiation trajectories and maintaining the chronic inflammatory microenvironment in PsO.

To further delineate the immune and stromal microenvironments in PsO, we performed independent subclustering of T cells and fibroblasts. Within the T cell compartment, three subtypes were identified: Th17 cells, CD8^+^ T cells, and Cytotoxic T cells. Although Th17 cells remained predominant in both groups, their proportion was reduced in PsO while CD8^+^ T cells markedly expanded, suggesting a shift from Th17 dominance toward cytotoxic infiltration. Notably, the CD8^+^ T cell subtype co-expressed exhaustion markers TIGIT and LAG3, reflecting chronic antigenic stimulation within psoriatic lesions and implying a more heterogeneous T cell response beyond the classical Th17-centric model (97). Within the fibroblast compartment, F2 Universal (Reticular) fibroblasts expanded in PsO, while immunoregulatory F3 FRC-like fibroblasts (marked by HLA-DRA, CD74, and APOE) were relatively reduced. The loss of this immunosuppressive stromal subset may compromise local immune homeostasis and facilitate the persistence of inflammatory infiltrates (98). A modest increase in F5 Schwann-like fibroblasts (NGFR^+^) may further contribute to neural-immune crosstalk in psoriatic skin (99). Together, these findings reveal that PsO lesions are characterized by coordinated remodeling of both the T cell and fibroblast compartments, with shifts toward cytotoxic and pro-inflammatory states that cooperate with KC dysfunction to sustain chronic inflammation.

This study has several limitations. First, the transcriptomic analyzes rely on public datasets derived from heterogeneous platforms. Although batch effects were corrected, residual confounding cannot be fully excluded and causal relationships require further confirmation (100). Second, the *in vitro* functional validation was conducted in the HaCaT keratinocyte cell line, which does not fully recapitulate the complex psoriatic microenvironment including immune cell infiltration and cytokine crosstalk, and the findings lack systematic validation *in vivo* PsO animal model. Third, the IHC validation, while providing protein-level evidence, was performed on a relatively small clinical cohort, which limits statistical power and generalizability across PsO subtypes and disease severities. In addition, the patient samples used for IHC in the current study were obtained from archived paraffin blocks without complete accompanying clinical records. Therefore, we are unable to retrospectively determine the precise PASI scores for each subject. The multicenter, prospective, long-term studies with well-documented clinical phenotyping be conducted to evaluate the potential of SLC25A4 as a biomarker linked to PsO severity or subtypes. Meanwhile, PsO animal models should be employed to elucidate how SLC25A4 and related genes regulate disease progression via metabolic-immune networks, and to explore therapeutic strategies targeting these biomarkers, thereby advancing the clinical translation of metabolic interventions for PsO.

## 5 Conclusions

In this study, we identified three lactate metabolism–related genes (GOT2, PYGL, and SLC25A4) as potential biomarkers linking metabolic reprogramming, immune dysregulation, and keratinocyte pathology in PsO. Among them, SLC25A4 appears particularly pivotal: its downregulation in PsO correlated with immune-activation, suggesting a role in restraining inflammation. Functionally, SLC25A4 is essential for keratinocyte proliferation and survival, as its knockdown induced apoptosis. Moreover, its involvement in the JAK–STAT pathway, its integration into regulatory networks centered on NEAT1/miR-23a-3p and GATA3, and its druggability potential through binding to small molecules (e.g., clodronate), SLC25A4 may not only as a diagnostic marker but also represent a promising mitochondria-related therapeutic target for metabolic–immune intervention in PsO.

## Data Availability

The original contributions presented in the study are included in the article/**Supplementary Material**. Further inquiries can be directed to the corresponding author.

## Supplementary material

Supplementary Figure 1 Enrichment analysis and PPI network construction. **(A)** Results of GO enrichment analysis. The left part is a circular graph of GO enrichment analysis. The inner circle represents the significance of the pathway, with higher values indicating greater significance. The color of the bar graph is the z - score, where blue represents down - regulated pathways and green represents up - regulated pathways. The outer circle shows a scatter plot of the gene expression levels in each pathway. The right figure shows the ID of the GO Term and the corresponding description. **(B)** Results of KEGG enrichment analysis. In the figure, the depth of the color represents the magnitude of logFC. The darker the color, the larger the logFC. Green indicates up - regulated genes and red indicates down - regulated genes. Different colored squares represent different pathways. The more genes enriched in a pathway, the larger the colored square. **(C)** Construction of the PPI network.

Supplementary Figure 2 Functional analysis of biomarkers. **(A)** Analysis of the tissue localization of GOT2, PYGL, and SLC25A4. The abscissa represents the tissue type, and the ordinate represents the gene expression score. **(B)** Analysis of the correlation of biomarkers. *p<0.05, **p<0.01, ***p<0.001. **(C)** Disease prediction of biomarkers.

Supplementary Figure 3 In addition to single - cell data. Before **(A)** and after **(B)** quality control of single - cell data. **(C)** Screening of highly variable genes, with highly variable genes shown in red. **(D)** PCA dimensionality reduction plot. **(E)** PCA scree plot. **(F)** UMAP dimensionality reduction plot for cell cluster clustering, with different colors representing different clusters.

Supplementary Figure 4 Quality control and clustering process for T cell subclustering. **(A)** PCA plot after Harmony batch correction. **(B)** Highly variable gene identification showing 2,000 selected HVGs. **(C)** Elbow plot for PC selection. **(D)** JackStraw plot confirming PC significance. **(E)** UMAP plot of raw unsupervised clustering. **(F)** tSNE plot of raw unsupervised clustering. **(G)** Bubble plot of marker gene expression across raw clusters. **(H)** Clustree analysis supporting selection of resolution = 0.1.

Supplementary Figure 5 Quality control and clustering process for fibroblast subclustering. **(A)** PCA plot after Harmony batch correction. **(B)** Highly variable gene identification showing 2,000 selected HVGs. **(C)** Elbow plot for PC selection. **(D)** JackStraw plot confirming PC significance. **(E)** UMAP plot of raw unsupervised clustering. **(F)** tSNE plot of raw unsupervised clustering. **(G)** Bubble plot of marker gene expression across raw clusters. **(H)** Clustree analysis supporting selection of resolution = 0.1.

Supplementary Table 1 LRGs information.

Supplementary Table 2 PsO-DEGs information.

Supplementary Table 3 Results of GO enrichment analysis.

Supplementary Table 4 Results of KEGG enrichment analysis.

Supplementary Table 5 Heterogeneity test.

Supplementary Table 6 Horizontal pleiotropic test.

Supplementary Table 7 Results of GSEA enrichment analysis of GOT2.

Supplementary Table 8 Results of GSEA enrichment analysis of PYGL.

Supplementary Table 9 Results of GSEA enrichment analysis of SLC25A4.

Supplementary Table 10 Correlation analysis of biomarkers and immune cells.

Supplementary Table 11 Analysis of chemokine correlation.

Supplementary Table 12 Analysis of immunosuppressant correlation.

Supplementary Table 13 Analysis of immunostimulatory factor correlation.

Supplementary Table 14 Analysis of chemokine receptor correlation.

Supplementary Table 15 Analysis of MHC correlation.

Supplementary Table 16 Key miRNA information.

Supplementary Table 17 Related long non - coding RNA information.

Supplementary Table 18 Related circular RNA information.

Supplementary Table 19 Drug prediction analysis.

## Glossary

ANN: artificial neural network
BP: biological process
CC: cellular component
CCK-8: Cell Counting Kit-8
CI: confidence interval
CTD: Comparative Toxicogenomics Database
DC: dendritic cell
DE-LRGs: differentially expressed lactate metabolism-related genes
DEGs: differentially expressed genes
FBS: fetal bovine serum
FDR: false discovery rate
GEO: Gene Expression Omnibus
GGI: gene–gene interaction
GO: Gene Ontology
GSEA: gene set enrichment analysis
GWAS: genome-wide association study
HPA: Human Protein Atlas
HVGs: highly variable genes
IFN-γ: interferon gamma
IL-23: interleukin 23
IL-4: interleukin 4
IVW: inverse variance weighted
JAK: Janus kinase
KC: keratinocyte
KCs: keratinocytes
KEGG: Kyoto Encyclopedia of Genes and Genomes
LASSO: least absolute shrinkage and selection operator
LRGs: lactate metabolism-related genes
MF: molecular function
MIF: Macrophage Migration Inhibitory Factor
MNC: Maximum Neighborhood Component
MR: Mendelian randomization
MSigDB: Molecular Signatures Database
NES: normalized enrichment score
NK: natural killer
NKT: natural killer T
NLRP3: NLR family pyrin domain containing 3
OR: odds ratio
PC: principal component
PCA: principal component analysis
PPI: protein–protein interaction
PsO: psoriasis
ROC: receiver operating characteristic
ROS: reactive oxygen species
SD: standard deviation
SHP2: Src homology region 2-containing protein tyrosine phosphatase-2
SNP: single-nucleotide polymorphism
STAT: signal transducer and activator of transcription
STAT3: signal transducer and activator of transcription 3
STRING: Search Tool for the Retrieval of Interacting Genes
SVM-RFE: support vector machine-recursive feature elimination
UMAP: uniform manifold approximation and projection
WGCNA: Weighted Gene Co-expression Network Analysis.
