# Supplementary figures and images for: Integrating bulk RNA-seq, Mendelian randomization and single-cell RNA-seq to elucidate the roles of lactate metabolism related markers-SLC25A4 and keratinocyte in the pathogenesis of psoriasis
Source: Front Immunol. 2026 May 29;17:1842933. doi: 10.3389/fimmu.2026.1842933 (PMC13260234; doi:10.3389/fimmu.2026.1842933)

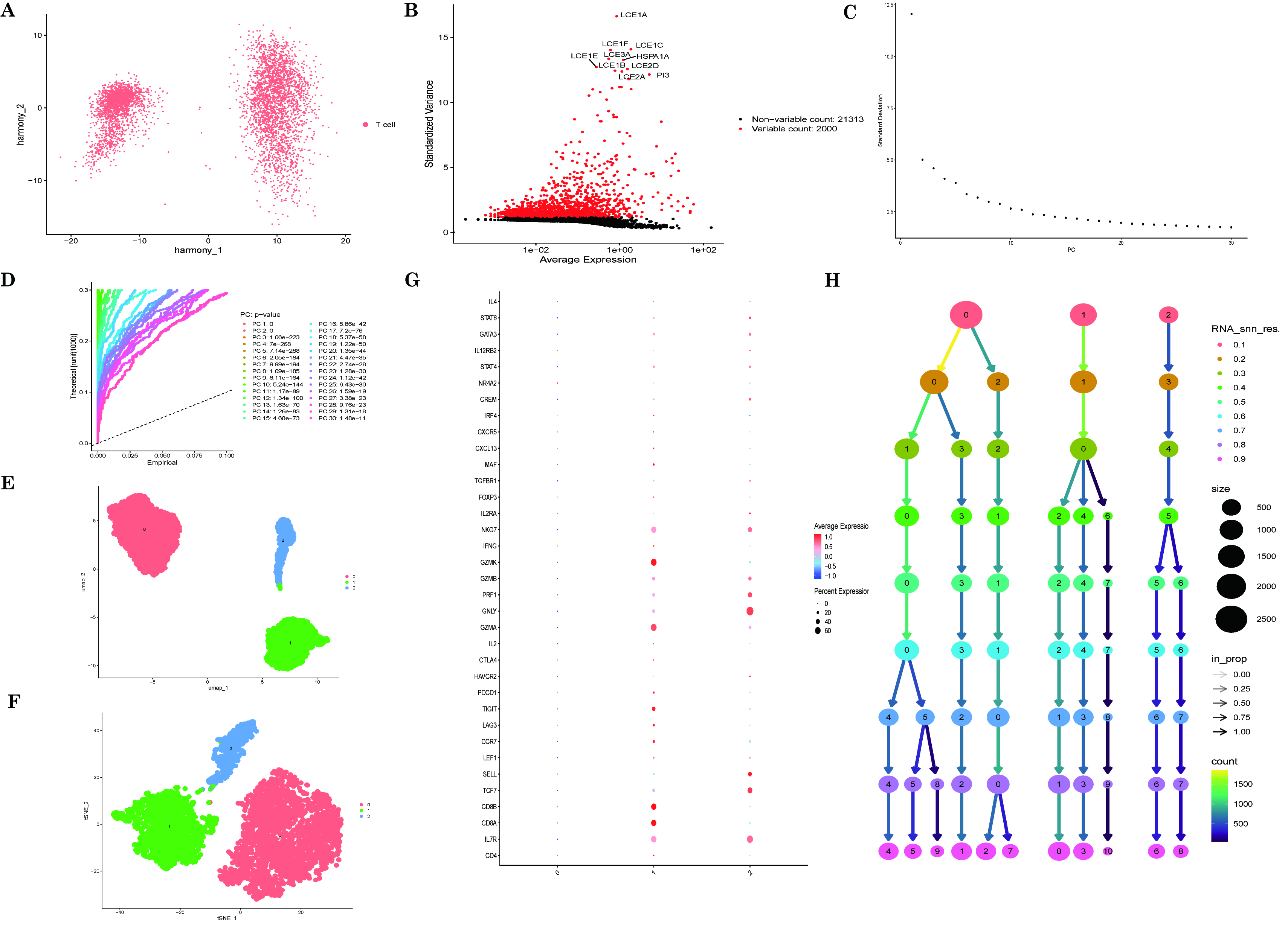

Supplement: Supplementary Figure 1 — Enrichment analysis and PPI network construction. (A) Results of GO enrichment analysis. The left part is a circular graph of GO enrichment analysis. The inner circle represents the significance of the pathway, with higher values indicating greater significance. The color of the bar graph is the z - score, where blue represents down - regulated pathways and green represents up - regulated pathways. The outer circle shows a scatter plot of the gene expression levels in each pathway. The right figure shows the ID of the GO Term and the corresponding description. (B) Results of KEGG enrichment analysis. In the figure, the depth of the color represents the magnitude of logFC. The darker the color, the larger the logFC. Green indicates up - regulated genes and red indicates down - regulated genes. Different colored squares represent different pathways. The more genes enriched in a pathway, the larger the colored square. (C) Construction of the PPI network. [file SupplementaryFile1.zip › Supplementary materials/Figure S4.tif]

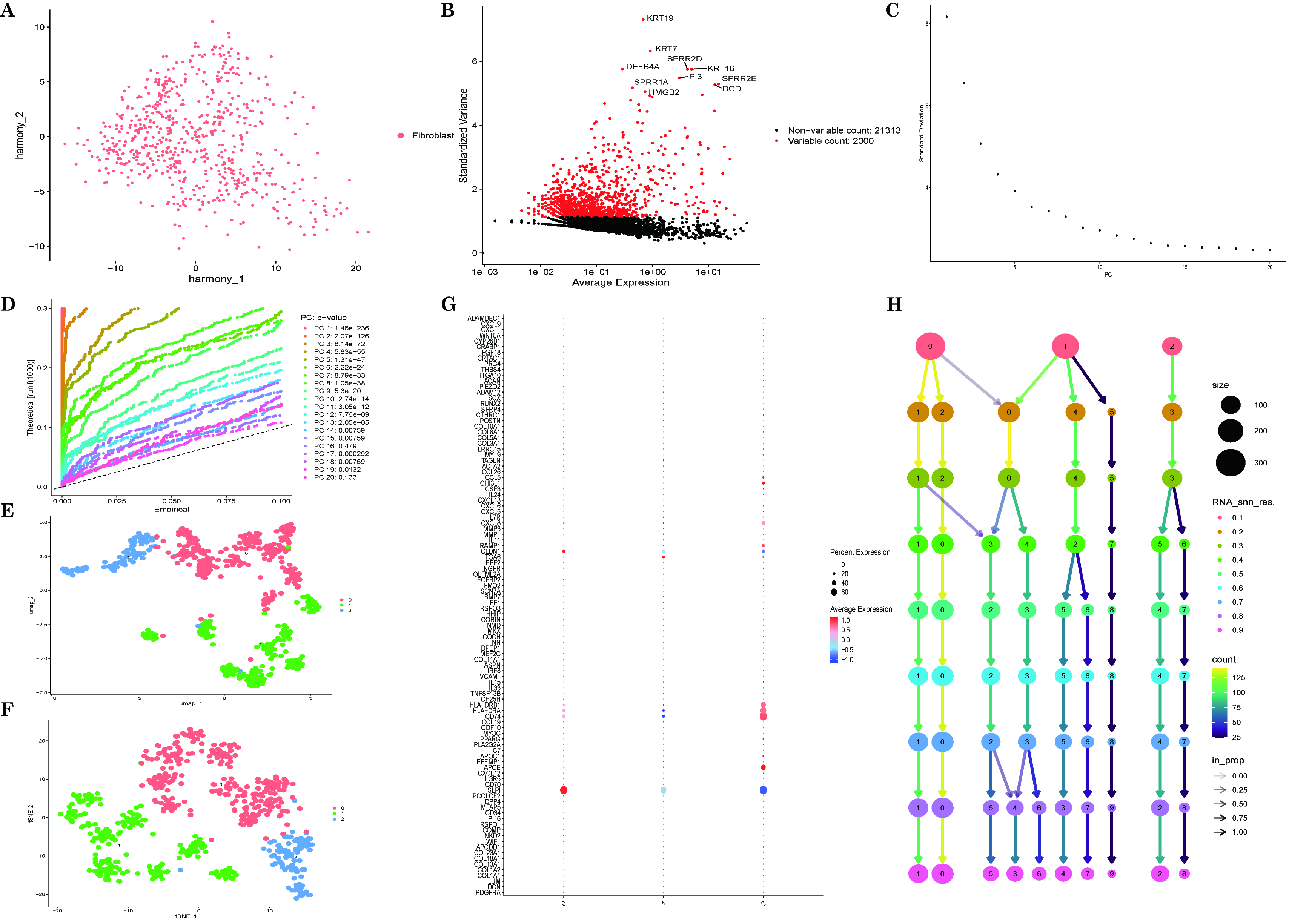

Supplement: Supplementary Figure 1 — Enrichment analysis and PPI network construction. (A) Results of GO enrichment analysis. The left part is a circular graph of GO enrichment analysis. The inner circle represents the significance of the pathway, with higher values indicating greater significance. The color of the bar graph is the z - score, where blue represents down - regulated pathways and green represents up - regulated pathways. The outer circle shows a scatter plot of the gene expression levels in each pathway. The right figure shows the ID of the GO Term and the corresponding description. (B) Results of KEGG enrichment analysis. In the figure, the depth of the color represents the magnitude of logFC. The darker the color, the larger the logFC. Green indicates up - regulated genes and red indicates down - regulated genes. Different colored squares represent different pathways. The more genes enriched in a pathway, the larger the colored square. (C) Construction of the PPI network. [file SupplementaryFile1.zip › Supplementary materials/Figure S5.tif]

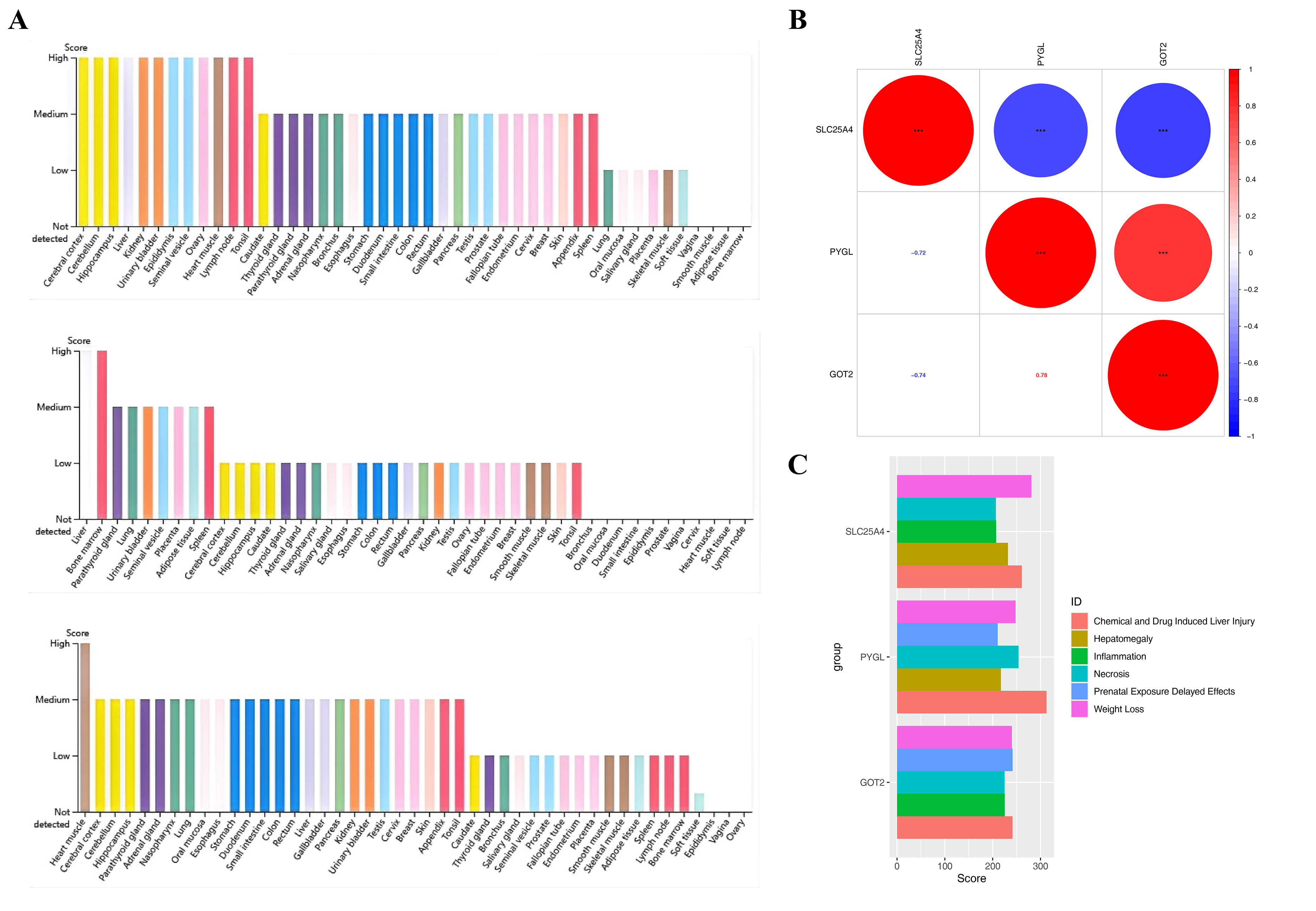

Supplement: Supplementary Figure 1 — Enrichment analysis and PPI network construction. (A) Results of GO enrichment analysis. The left part is a circular graph of GO enrichment analysis. The inner circle represents the significance of the pathway, with higher values indicating greater significance. The color of the bar graph is the z - score, where blue represents down - regulated pathways and green represents up - regulated pathways. The outer circle shows a scatter plot of the gene expression levels in each pathway. The right figure shows the ID of the GO Term and the corresponding description. (B) Results of KEGG enrichment analysis. In the figure, the depth of the color represents the magnitude of logFC. The darker the color, the larger the logFC. Green indicates up - regulated genes and red indicates down - regulated genes. Different colored squares represent different pathways. The more genes enriched in a pathway, the larger the colored square. (C) Construction of the PPI network. [file SupplementaryFile1.zip › Supplementary materials/Figure S2.tif]

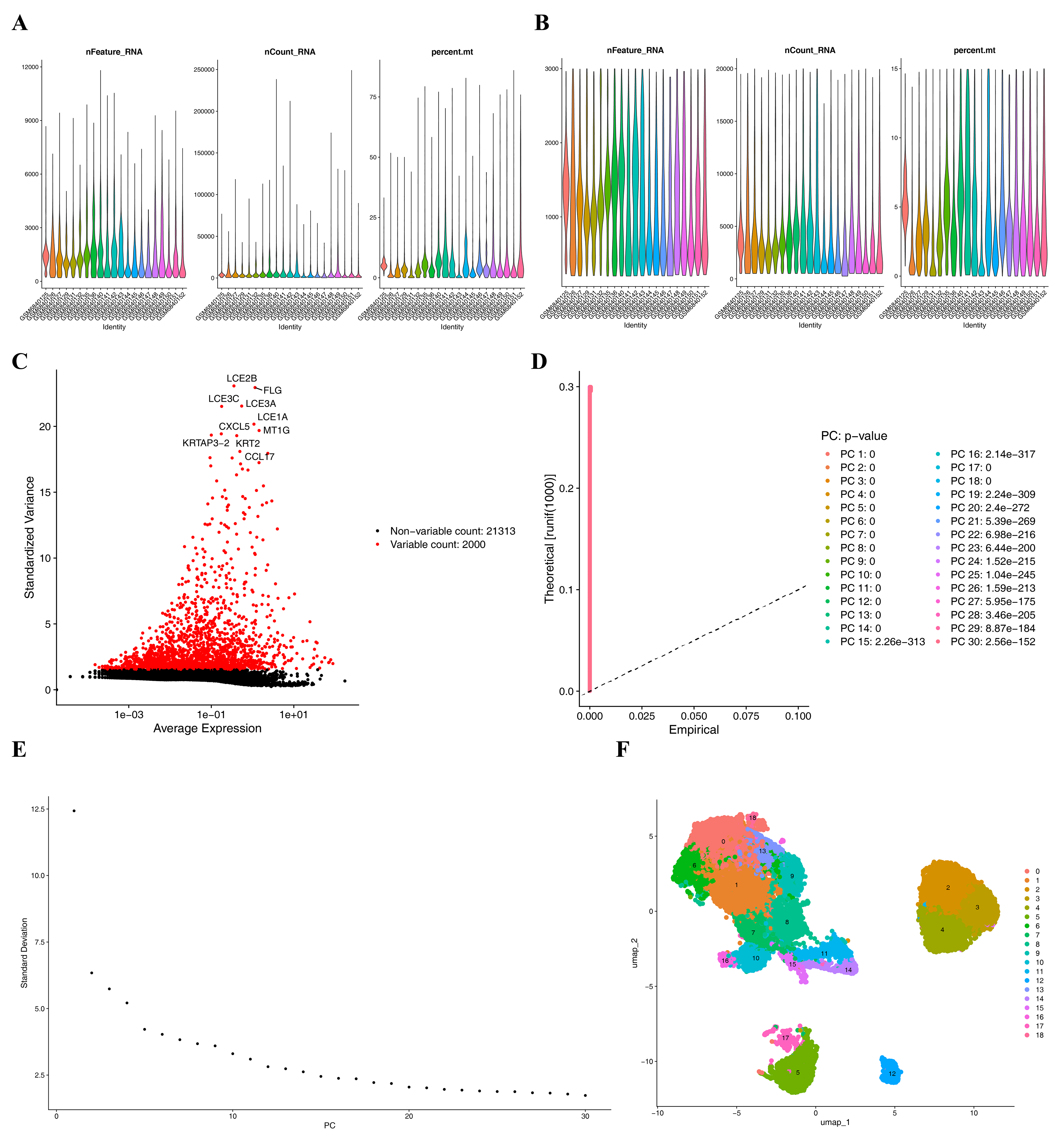

Supplement: Supplementary Figure 1 — Enrichment analysis and PPI network construction. (A) Results of GO enrichment analysis. The left part is a circular graph of GO enrichment analysis. The inner circle represents the significance of the pathway, with higher values indicating greater significance. The color of the bar graph is the z - score, where blue represents down - regulated pathways and green represents up - regulated pathways. The outer circle shows a scatter plot of the gene expression levels in each pathway. The right figure shows the ID of the GO Term and the corresponding description. (B) Results of KEGG enrichment analysis. In the figure, the depth of the color represents the magnitude of logFC. The darker the color, the larger the logFC. Green indicates up - regulated genes and red indicates down - regulated genes. Different colored squares represent different pathways. The more genes enriched in a pathway, the larger the colored square. (C) Construction of the PPI network. [file SupplementaryFile1.zip › Supplementary materials/Figure S3.tif]

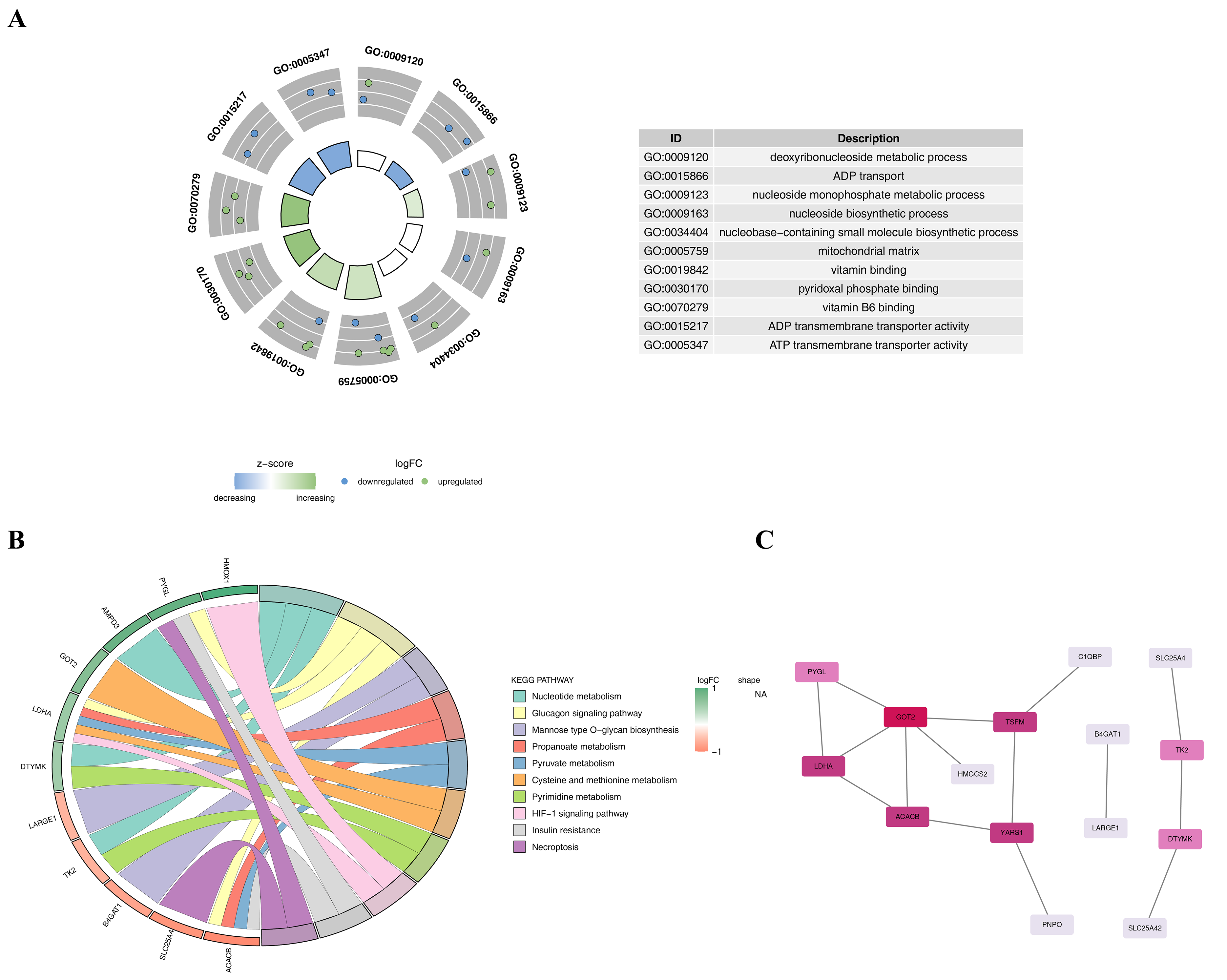

Supplement: Supplementary Figure 1 — Enrichment analysis and PPI network construction. (A) Results of GO enrichment analysis. The left part is a circular graph of GO enrichment analysis. The inner circle represents the significance of the pathway, with higher values indicating greater significance. The color of the bar graph is the z - score, where blue represents down - regulated pathways and green represents up - regulated pathways. The outer circle shows a scatter plot of the gene expression levels in each pathway. The right figure shows the ID of the GO Term and the corresponding description. (B) Results of KEGG enrichment analysis. In the figure, the depth of the color represents the magnitude of logFC. The darker the color, the larger the logFC. Green indicates up - regulated genes and red indicates down - regulated genes. Different colored squares represent different pathways. The more genes enriched in a pathway, the larger the colored square. (C) Construction of the PPI network. [file SupplementaryFile1.zip › Supplementary materials/Figure S1.tif]
